# Insulin-Like Growth Factor Binding Protein-3 Exerts Its Anti-Metastatic Effect in Aerodigestive Tract Cancers by Disrupting the Protein Stability of Vimentin

**DOI:** 10.3390/cancers13051041

**Published:** 2021-03-02

**Authors:** Huong Thuy Le, Ho Jin Lee, Jaebeom Cho, Hye-Young Min, Ji-Sun Lee, Su-Jae Lee, Ho-Young Lee

**Affiliations:** 1 Creative Research Initiative Center for Concurrent Control of Emphysema and Lung Cancer, College of Pharmacy and Research Institute of Pharmaceutical Sciences, Seoul National University, Seoul 08826, Korea; lethuyhuong@tdtu.edu.vn (H.T.L.); hojin1122@snu.ac.kr (H.J.L.); gslife@snu.ac.kr (J.C.); snoopy77@snu.ac.kr (H.-Y.M.); jslee5995@hanmail.net (J.-S.L.); 2 Department of Life Science, Research Institute for Natural Sciences, Hanyang University, Seoul 04763, Korea; sj0420@hanyang.ac.kr

**Keywords:** insulin-like growth factor binding protein-3, vimentin, metastasis

## Abstract

**Simple Summary:**

Local invasion and distal metastasis are the main causes of cancer-related death and the poor prognosis of patients with aerodigestive tract cancers. Therefore, understanding the biology of invasion and metastasis is important for the development of effective therapeutic strategies. The present study shows that insulin-like growth factor binding protein-3 (IGFBP-3) inhibits the migration and invasion of non-small cell lung cancer (NSCLC) and head and neck squamous cell carcinoma (HNSCC) cells in vitro and the development of metastasized tumors in vivo. Mechanistic studies suggest vimentin as a cellular target for the antimetastatic effect of IGFBP-3. These results contribute to a better understanding on the regulation of metastasis of cancer cells, providing the rationale to utilize IGFBP-3 as an effective therapeutic strategy targeting migration and metastasis of aerodigestive tract cancers.

**Abstract:**

The proapoptotic, antiangiogenic, and antimetastatic activities of insulin-like growth factor binding protein-3 (IGFBP-3) through IGF-dependent or -independent mechanisms have been suggested in various types of human cancers. However, a mechanistic explanation of and downstream targets involved in the antimetastatic effect of IGFBP-3 is still lacking. In this study, by applying various in vitro and in vivo models, we show that IGFBP-3 suppresses migration and invasion of human head and neck squamous carcinoma (HNSCC) and non-small cell lung cancer (NSCLC) cells. Silencing IGFBP-3 expression elevated the migration and invasion of NSCLC and HNSCC cells in vitro and their local invasion and metastasis in vivo, whereas overexpression of IGFBP-3 decreased such prometastatic changes. Local invasion of 4-nitroquinoline-1-oxide (4-NQO)-induced HNSCC tumors was consistently significantly potentiated in *Igfbp3* knockout mice compared with that in wild-type mice. Mechanistically, IGFBP-3 disrupted the protein stability of vimentin via direct binding and promoting its association with the E3 ligase FBXL14, causing proteasomal degradation. The C-terminal domain of IGFBP-3 and the head domain of vimentin are essential for their interaction. These results provide a molecular framework for IGFBP-3′s IGF-independent antimetastatic and antitumor activities.

## 1. Introduction

Aerodigestive tract cancers, including non-small cell lung cancer (NSCLC) and head and neck squamous cell carcinoma (HNSCC), are the leading cause of cancer-related deaths worldwide [1,2]. Invasion and metastasis are associated with poor prognosis of patients with these cancers [3,4,5,6]; however, there is no available therapeutic option to cure invasive or metastatic tumors because of the limited understanding of the underlying mechanisms due to the complexity of the biology of metastasis.

Epithelial-mesenchymal transition (EMT) and its reverse process, the mesenchymal-epithelial transition (MET), play a critical role in the invasion and metastasis of cancer cells [7]. EMT is characterized by the downregulation of epithelial proteins, such as E-cadherin, and the elevation of several mesenchymal markers such as vimentin and fibronectin [8]. Among several EMT-related markers, vimentin is an intermediate filament that mediates cell motility and polarity through cytoskeletal rearrangement [9]. Suppression of vimentin expression was found to block the migration and adhesion of cancer cells [10]. In addition, previous studies have shown the impact of vimentin expression as a poor prognosis marker for patients with NSCLC or HNSCC [11,12,13,14]. Several transcription factors, including forkhead box proteins, ZNF703, PRX1, Snail, Twist1, Zeb1, Slug, and Smad interacting protein-1 (SIP1), have been reported to regulate vimentin expression [8,9,15,16]. A recent study demonstrated negative regulation of vimentin stability by an E3 ligase RNF208 [17]. However, the detailed mechanisms underlying the regulation of vimentin are largely unknown [9].

The insulin-like growth factor (IGF) axis plays an important role in cell proliferation, growth, survival, and longevity [18]. Aberrant IGF signaling is associated with the development and progression of various human cancers [18]. Insulin-like growth factor binding proteins (IGFBPs) have shown antitumor activity by binding to free IGFs (IGF1 or IGF2) and blocking their ability to activate type I insulin-like growth factor receptor (IGF-1R) [19]. Seven insulin-like growth factor binding proteins (IGFBPs; IGFBP-1-IGFBP-7) have been identified; among them, IGFBP-3 is a predominant form of IGFBP in circulation [19]. IGFBP-3 has shown antitumor activities by regulating proliferation, migration, adhesion, and/or angiogenesis [20,21,22,23,24], and dysregulation of IGFBP-3 has been associated with various human cancers [19]. In addition to regulating the bioavailability of IGFs, IGFBP-3 has been shown to interact with non-IGF cellular proteins, such as low-density lipoprotein receptor-related protein 1 (LRP1), retinoid X receptor-α (RXR-α), and Nur77 [19,25,26]. Although the interaction of IGFBP-3 with non-IGF proteins contributes to the proapoptotic role of IGFBP-3, the association of IGFBP-3 with other cellular proteins and its contribution to IGFBP-3′s intrinsic roles remain to be elucidated. Moreover, the mechanism underlying antimetastatic effect of IGFBP-3 has been poorly investigated.

Here we demonstrate a novel mechanism underlying antimetastatic effect of IGFBP-3 in vitro and in vivo. Silencing of IGFBP-3 expression increased the migration and invasion of HNSCC and NSCLC cells by upregulating the EMT program, and overexpression of IGFBP-3 reversed such phenotype changes. Consistently, upregulation of the metastasized tumor formation was observed in systemic *Igfbp3* knockout (KO) mice, wherein tongue tumors were induced by 4-nitroquinoline-1-oxide (4-NQO) [27], and wild-type mice in which xenograft tumors were established by orthotopic or subcutaneous injection of human HNSCC or NSCLC cells with knocked down IGFBP-3 expression. Further mechanistic studies revealed that IGFBP-3 negatively regulates EMT phenotypes of NSCLC and HNSCC cells and suppresses their migratory activities in an IGF-independent manner by directly binding to vimentin and inducing its degradation through the E3 ligase FBXL14-mediated proteasome machinery. These results collectively suggest the role of IGFBP-3 as a negative regulator of the EMT program for metastasis through vimentin destabilization in cooperation with FBXL14. In addition, downregulation of vimentin through utilizing IGFBP-3 can be a novel strategy to block EMT and metastasis in NSCLC and HNSCC.

## 2. Results

### 2.1. IGFBP-3 Inhibits the Migratory and Invasive Abilities of NSCLC and HNSCC Cells by Downregulating EMT Phenotypes

**Figure 1:**
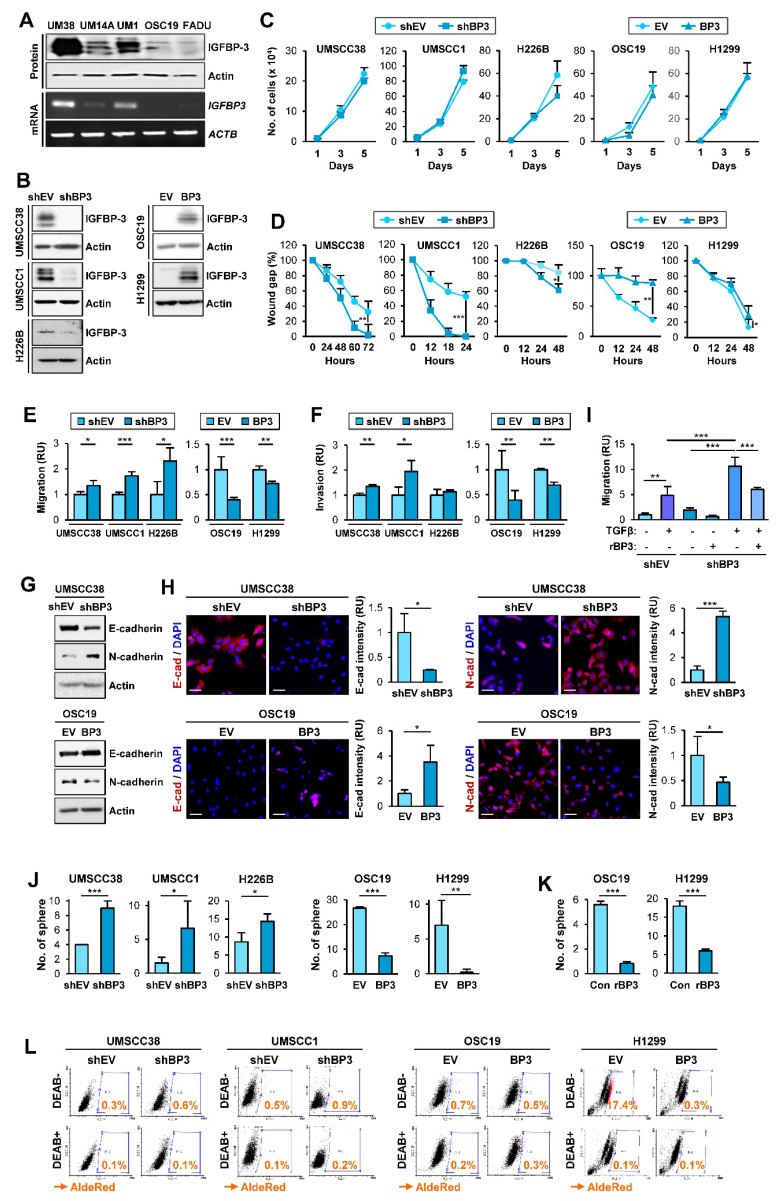
IGFBP-3 inhibits the acquisition of EMT and CSC-like phenotypes in HNSCC and NSCLC cells. (**A**,**B**) Western blot (WB) (**A**,**B**) and RT-PCR (**A**) analyses of IGFBP-3 expression in the indicated HNSCC cell lines (**A**) and HNSCC and NSCLC cell lines wherein IGFBP-3 expression was either silenced of enforced by stable transfection with shRNA or expression vector (**B**). (**C**) Effect of IGFBP-3 on cell proliferation was accessed by cell counting assay. (**D**–**F**) Effects of IGFBP-3 expression on the migration and invasion of the indicated cancer cells evaluated by a scratch assay (**D**) and by the Transwell migration (**E**) and invasion (**F**) assays. (**G**,**H**) WB (**G**) and IF (**H**) analyses for the protein expression of E-cadherin and N-cadherin in the indicated cancer cells with manipulation of IGFBP-3 expression. Quantification of the fluorescence intensity was analyzed by ImageJ software (**H**). Scale bars: 50 μm (**H**). (**I**) UMSCC38-shEV and UMSCC38-shBP3 cells unstimulated or stimulated with TGF-β (10 ng/mL) in the absence or presence of recombinant IGFBP-3 (10 μg/mL) for 48 h were subjected to the Transwell migration assay (**J**–**L**) Regulation of sphere formation (**J**,**K**) and ALDH activity (**L**) by manipulation of IGFBP-3 expression (**J**,**L**) or treatment with recombinant IGFBP-3 protein (**K**). The bar represents mean ± SD. * *p* < 0.05, ** *p* < 0.01, and *** *p* < 0.001, as determined by the two-tailed Student’s *t*-test compared with the corresponding control (**C**–**F**,**H**,**J**,**K**) and one-way ANOVA followed by Dunnett’s post-hoc test (**I**). UM38: UMSCC38; UM14A: UMSCC14A; UM1: UMSCC1; OSC19: OSC19-Luc. EV: empty vector; BP3: IGFBP-3.

Given IGFBP-3 overexpression suppresses the angiogenic and metastatic activities of NSCLC cells [22,23,24,28], we assessed whether IGFBP-3 regulates the proliferative, migratory, and invasive activities of HNSCC and NSCLC cells and investigated how IGFBP-3 exhibits such activities. We performed a series of in vitro experiments to assess the effects of IGFBP-3 on the proliferative, migratory, and invasive activities of HNSCC and NSCLC cells. To this end, we first evaluated the mRNA and protein expression of IGFBP-3 in several HNSCC cell lines and selected cell lines with high (UMSCC38, UMSCC1) or low (OSC19-Luc) levels of IGFBP-3 expression (Figure 1A). We established UMSCC38 and UMSCC1, in which IGFBP-3 expression was silenced by stable transfection with shRNAs [UMSCC38-shBP3 (UM38-shBP3) and UMSCC1-shBP3], and OSC19-Luc cells with forced overexpression of IGFBP-3 (OSC19-BP3) (Figure 1B). We also selected NSCLC cell lines with high (H226B) or low (H1299) levels of IGFBP-3 expression [29] and established their counterparts (H226B-shBP3 and H1299-BP3) by stable transfection with shRNAs or by forced overexpression of IGFBP-3 (Figure 1B). We then determined the effects of IGFBP-3 expression on the proliferative and migratory phenotypes in the selected HNSCC and NSCLC cell lines. These cell lines with manipulation of IGFBP-3 expression showed minimal difference in proliferation compared with their corresponding control cells (Figure 1C). In contrast, when migratory activities were analyzed using a scratch assay, UMSCC38-shBP3, UMSCC1-shBP3, and H226-shBP3 cells closed the wound faster than the corresponding control cells (UMSCC38-shEV, UMSCC1-shEV, and H226B-shEV), whereas OSC19-BP3 and H1299-BP3 cells showed significantly delayed wound closure compared to their corresponding cells (Figure 1D). Consistently, UMSCC38-shBP3, UMSCC1-shBP3, and H226B-shBP3 cells showed significantly greater migration (Figure 1E) and invasion (Figure 1F) in Transwells compared with their corresponding cells, while the activities of OSC19-BP3 and H1299-BP3 cells were significantly reduced compared with those of their control cells. These results suggest the regulatory role of IGFBP-3 in the migration and invasion of HNSCC and NSCLC cells.

Given that the acquisition of the EMT program is closely associated with the migratory and invasive phenotypes [8], we investigated the effects of IGFBP-3 on the expression of EMT-associated markers. Western blot (Figure 1G) and immunofluorescence (IF) (Figure 1H) analyses revealed that silencing IGFBP-3 expression mediated the upregulation of N-cadherin and concomitant downregulation of E-cadherin. Conversely, overexpression of IGFBP-3 markedly induced E-cadherin expression and suppressed N-cadherin expression. We confirmed the role of IGFBP-3 in the EMT process mediated by a known EMT inducer, transforming growth factor-β (TGF-β) [30]. Because cancer cell migration is a typical phenotype of the EMT program [31], we determined the role of IGFBP-3 in TGF-β-induced migration. While both UMSCC38-shEV cells and UMSCC38-shBP3 cells showed increased migration in response to TGF-β treatment, UMSCC38-shBP3 cells showed significantly greater degree of migration than did the UMSCC38-shEV cells (Figure 1I). Moreover, the basal and TGF-β-enhanced migration of UMSCC38-shBP3 cells was significantly suppressed by treatment with recombinant IGFBP-3 protein. We also assessed the effect of IGFBP-3 on the EMT program by performing in silico analysis. Analysis using the The Cancer Genome Atlas (TCGA) dataset and datasets available in the Gene Expression Omnibus (GEO) database revealed the inverse correlation of the *IGFBP3* (encoding the IGFBP-3 protein) mRNA expression with a mesenchymal marker *VIM* (encoding the vimentin protein) and the positive correlation between *IGFBP3* mRNA and *CDH1* mRNA (encoding the E-cadherin protein) in tumors of patients with NSCLC (Figure S1), confirming the regulation of the EMT program by IGFBP-3.

Based on the close association between the activation of the EMT program and the acquisition of cancer stem cell (CSC) properties [32], we next examined the effects of IGFBP-3 on the regulation of CSC phenotypes. Compared with their corresponding control cells, HNSCC and NSCLC cells carrying shRNA-mediated IGFBP-3 ablation displayed increased sphere-forming capacities while those carrying IGFBP3 overexpression had reduced sphere-forming capacities (Figure 1J). We further confirmed that treatment with recombinant IGFBP-3 significantly suppressed the sphere-forming abilities of HNSCC and NSCLC cells (Figure 1K). Because elevation of ALDH activity has been considered a CSC marker in various cancer types [33], we evaluated the effect of IGFBP-3 on the regulation of ALDH activity by using a red fluorescent ALDH substrate (Aldered 588-A) [34]. As shown in Figure 1L, depletion of endogenous IGFBP-3 expression elevated the ALDH^high^ population, whereas ectopic IGFBP-3 expression resulted in a marked reduction in the ALDH^high^ population. Together, these results indicate that IGFBP-3 has the capacity to negatively regulate the EMT program in HNSCC and NSCLC cells.

### 2.2. IGFBP-3 Inhibits the Metastatic Potential of HNSCC and NSCLC Cells

**Figure 2:**
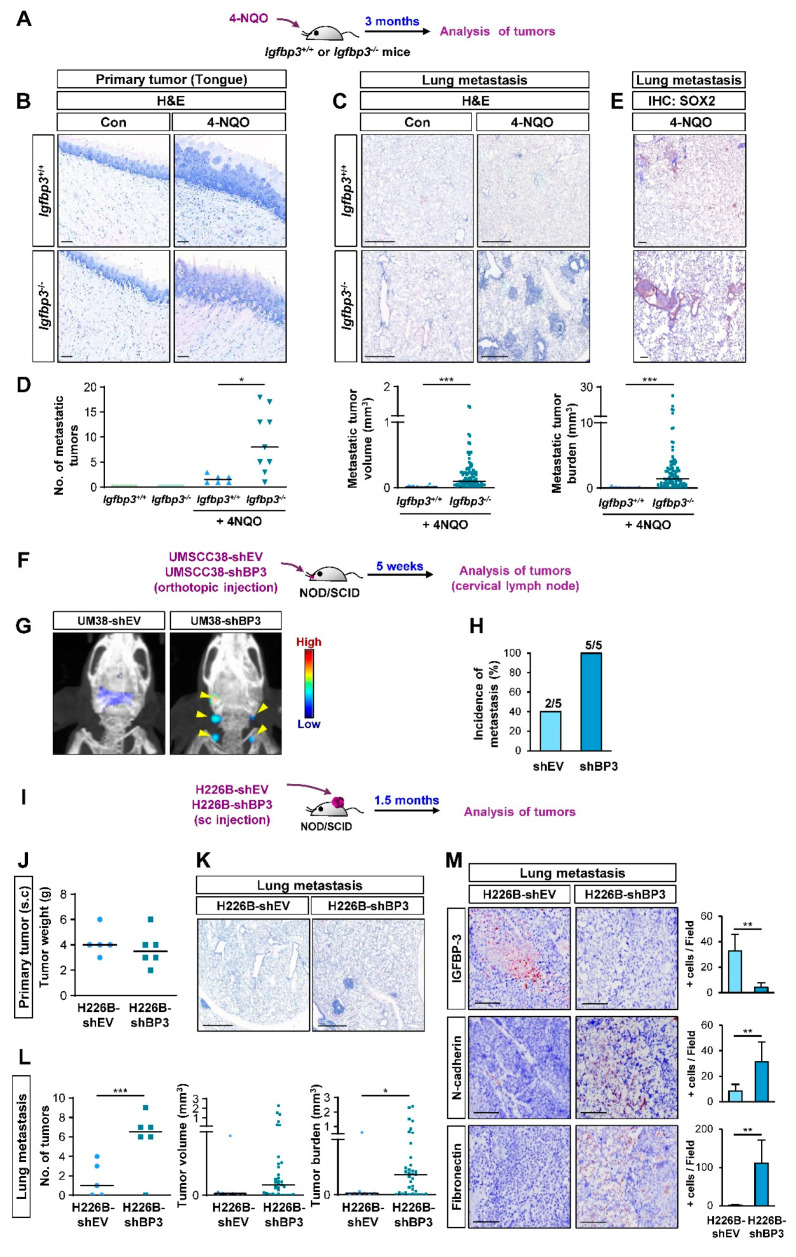
IGFBP-3 suppresses metastasis in vivo. (**A**) Schematic diagram illustrating the protocol to assess the impact of IGFBP-3 expression in metastasis of mouse HNSCC using the 4-NQO-induced HNSCC tumorigenesis model (*Igfbp3^+/+^* Con: *n* = 6; *Igfbp3^+/+^* 4-NQO: *n* = 7; *Igfbp3^−/−^* Con: *n* = 6; *Igfbp3^−/−^* 4-NQO: *n* = 9). (**B**,**C**) Primary (**B**) and metastatic (**C**) tumor formation in 4-NQO-administered mice was determined by H&E staining of the tongue (**B**) and lung (**C**) tissues. Scale bars: 25 μm (**B**), 1 mm (**C**). (**D**) Microscopic evaluation of the metastatic tumors in the lungs. (**E**) IHC analysis to determine the level of SOX2 expression in lung tissues. Scale bars: 25 μm. (**F**) Schematic diagram illustrating the protocol to assess the impact of IGFBP-3 expression in metastasis of human HNSCC using orthotopic tongue xenograft tumor model (*n* = 5 per group). (**G**) Representative IVIS images showing metastatic tumor formation in mice bearing UMSCC38-shBP3 tumor xenografts. (**H**) Microscopic evaluation showing increased metastasis in cervical lymph nodes in mice bearing UMSCC38-shBP3 cells compared with those in UMSCC38-shEV cells. Three cervical lymph nodes per mouse were evaluated. (**I**) Schematic diagram illustrating the protocol to assess the impact of IGFBP-3 expression in metastasis of human NSCLC using NSCLC xenograft tumor model (shEV: *n* = 5; shBP3: *n* = 6). (**J**) Changes in the weight of primary tumors between H226B-shEV and H226B-shBP3 tumor xenografts. (**K**,**L**) Upregulated metastatic tumor formation in mice bearing H226B-shBP3 tumor xenografts compared with those bearing 226B-shEV tumor xenografts, as determined by H&E staining of lung tissue sections (**K**) and microscopic evaluation of H&E-stained tissues (**L**). (**M**) The level of tumoral expression of N-cadherin and fibronectin in H226B-shEV and H226B-shBP3 xenograft tumors was determined by IHC analysis. Scale bars: 1 mm (**K**), 0.5 mm (**M**). The bar represents mean ± SD. * *p* < 0.05, ** *p* < 0.01, and *** *p* < 0.001, as determined by Mann-Whitney test (**D**,**L** (middle and right), **M**) and the two-tailed Student’s *t*-test (**L**, left) compared with the corresponding control. EV: empty vector; BP3: IGFBP-3.

We next assessed the impact of IGFBP-3 on the metastatic activities of HNSCC and NSCLC cells in vivo in mice by performing a series of animal experiments. We first employed a systemic *Igfbp3* knockout (KO) mouse model on an FVB/N background [35], wherein tongue tumors were established by exposure to 4-NQO, a water-soluble carcinogen that mainly generates tumors in the oral cavity [36,37], diluted in the drinking water, as previously described [27] (Figure 2A). Both WT and *Igfbp3* KO mice showed primary tongue tumor formation at 100% incidence after 4-NQO exposure for three months (Figure 2B). Analysis of the lung tissues revealed that, in contrast to previous reports [27,36], the 4-NQO-induced HNSCC in WT mice yielded some metastatic tumor nodules, presumably due to the high susceptibility of the FVB/N background to chemical-induced carcinogenesis [38,39] (Figure 2C,D). In contrast, *Igfbp3* KO mice with NQO administration showed significantly increased numbers, volume, and burden of tumor nodules (Figure 2C,D) with SOX2 expression (Figure 2E), a marker of squamous cell carcinoma [40]. Given only few carcinogens, such as methyl carbamate and N-nitroso-trischloroethylureas, have been known to develop squamous cell carcinoma in murine lungs [41], and a previous report also indicated that most lung tumors induced by 4-NQO were papillary adenomas [42]. Therefore, the tumoral expression of SOX2 indicates the lung metastasis of HNSCC tumors.

We further assessed the impact of IGFBP-3 on the metastatic activities of human HNSCC cells by utilizing orthotopic tongue tumor model of UMSCC38-shEV or UMSCC38-shBP3 in non-obese diabetic/severe combined immunodeficiency (NOD/SCID) mice (Figure 2F). Metastatic tumors, assessed by IVIS imaging using an MMP Sense 680 probe (Figure 2G) and histological evaluation (Figure 2H), revealed that mice bearing tongue tumors of UMSCC38-shBP3 experienced significantly increased local lymph node metastasis compared to those bearing UMSCC38-shEV tumors.

We next investigated the impact of IGFBP-3 expression on the metastatic potential of H226B-shEV and H226B-shBP3 cells in NOD/SCID mice (Figure 2I). When the primary and metastatic tumors in the mice were analyzed 1.5 months after the inoculation, the H226B-shEV and H226B-shBP3 xenograft tumors showed minimal differences in their growth as measured by tumor weights (Figure 2J). Analysis of H&E-stained lung tissues showed that mice harboring H226B-shBP3 tumors displayed markedly enhanced lung metastasis compared with those harboring H226B-shEV tumors (Figure 2K), as shown by the significantly increased number, volume, and burden of lung tumor nodules (Figure 2L). Moreover, immunohistochemistry (IHC) analyses revealed that the level of some EMT markers, such as N-cadherin and fibronectin, was elevated in H226B-shBP3 xenograft tumors compared with that of H226B-shEV xenograft tumors (Figure 2M). These results collectively indicate the role of IGFBP-3 in regulating metastasis of HNSCC and NSCLC cells.

### 2.3. IGFBP-3 Decreases Vimentin Protein Levels in an IGF-Independent Manner

**Figure 3:**
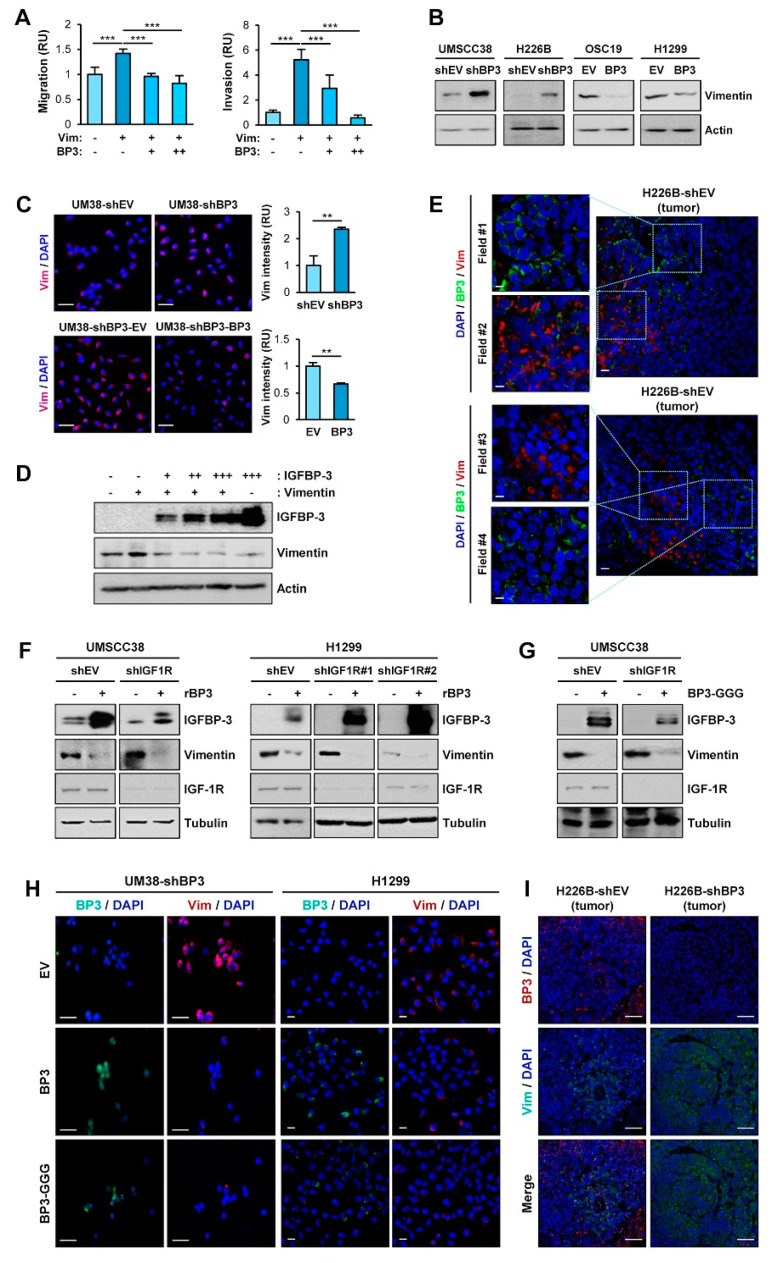
IGFBP-3 negatively regulates vimentin protein expression in an IGF-independent manner. (**A**) Transwell migration and invasion analyses of OSC19-Luc cells in which pEGFP-vimentin (Vim) was transiently transfected, either alone or together with increasing concentrations of expression vector carrying IGFBP-3 (BP3). (**B**,**C**) Western blot (WB) (**B**) and IF (**C**) analyses of vimentin expression in the indicated cancer cells with enforced overexpression or knockdown of IGFBP-3. Scale bars: 50 μm (**C**). (**D**) Dose-dependent inhibition of vimentin protein expression by overexpression of IGFBP-3. OSC19-Luc cells were transiently transfected with empty vectors (-, pEGFP and pCMV6), pEGFP-vimentin and increasing amounts of pCMV6-IGFBP-3 for 48 h. The expression levels of IGFBP-3 and vimentin were determined by WB analysis. (**E**) Inverse correlation between IGFBP-3 and vimentin expression in H226B-shEV tumor xenografts was determined by IF analysis. Scale bars: 10 μm (**left**), 20 μm (**right**). (**F–H**) WB (**F**,**G**) and IF(**H**) analyses of vimentin expression in the indicated cancer cells following treatment with recombinant IGFBP-3 protein (**F**) or transient transfection with empty vector [- (**G**) or EV (**H**)], pCMV6-IGFBP3 (BP3), or pCMV6-IGFBP3-GGG (BP3-GGG) (**G**,**H**). Scale bars: 50 μm (**H, left**), 20 μm (**H, right**). (**I**) IF analysis showing upregulation of vimentin expression in H226B-shBP3 tumors compared with H226B-EV tumors. Scale bars: 50 μm. The bar represents mean ± SD. ** *p* < 0.01 and *** *p* < 0.001, as determined by one-way ANOVA followed by Dunnett’s post-hoc test (**A**) and the two-tailed Student’s *t*-test compared with the corresponding control (**C**). Vim: vimentin; BP3: IGFBP-3; OSC19: OSC19-Luc; EV: empty vector.

Given the role of vimentin in EMT and cell migration and invasion [9], we assessed the implication of vimentin in the molecular mechanism by which IGFBP-3 inhibits the metastatic ability of HNSCC and NSCLC cells. OSC19-Luc cells were transiently transfected with empty vectors or expression vectors of vimentin, either alone or in combination with increasing amounts of IGFBP-3. Vimentin-induced migration and invasion of OSC19-Luc cells were significantly suppressed by IGFBP-3 in a dose-dependent manner (Figure 3A). We then assessed the effects of IGFBP-3 on vimentin expression. Western blot (Figure 3B) and IF (Figure 3C) analyses revealed that silencing of IGFBP-3 expression markedly elevated vimentin protein expression, while overexpression of IGFBP-3 downregulated vimentin expression. Overexpression of IGFBP-3 reduced vimentin protein levels in a dose-dependent manner (Figure 3D). Consistently, IF analysis of NSCLC and HNSCC tumors also showed an inverse correlation between IGFBP-3 and vimentin expression (Figure 3E).

Given that IGFBP-3 exerts cellular actions through both IGF-dependent and—independent manners [20,21], we additionally investigated whether the IGF signaling axis was implicated in the IGFBP-3-mediated regulation of vimentin expression. We found that treatment with recombinant IGFBP-3 significantly reduced vimentin in UMSCC38 and H1299 cells, either intact or loss of IGF1R expression through stable transfection with IGF1R shRNA (Figure 3F). Decreased vimentin expression was also observed in those UMSCC38 cells transfected with an expression vector carrying IGFBP-3 GGG (I56G, L80G, and L81G), a mutant IGFBP-3 with a reduced IGF binding affinity [43] (Figure 3G). IF staining also revealed a reduction in vimentin expression by overexpression of wild-type IGFBP-3 and the IGFBP-3 GGG mutant in UMSCC38-shBP3 (UM38-shBP3) and H1299 cells (Figure 3H). Moreover, upregulation of the vimentin level was observed in primary H226B-shBP3 tumors compared with H226B-shEV tumors (Figure 3I). Together, these results indicate that IGFBP-3 negatively regulates vimentin expression in an IGF-independent manner, and that vimentin is a cellular target for the antimetastatic effect of IGFBP-3.

### 2.4. IGFBP-3 Mediates the Deregulation of Vimentin Protein Stability

**Figure 4:**
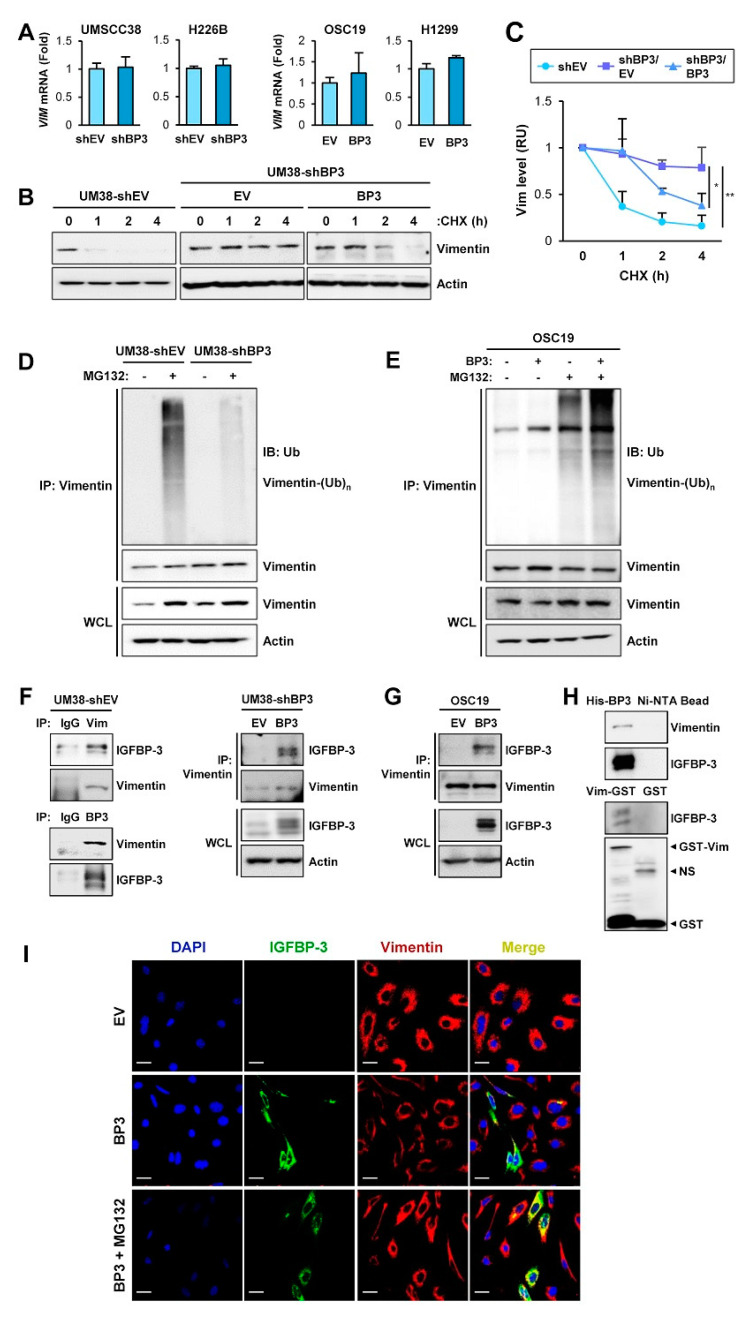
IGFBP-3 destabilizes vimentin via induction of ubiquitin-mediated proteasomal degradation of the vimentin protein. (**A**) Real-time PCR analysis of *VIM* mRNA levels in the indicated cancer cells with manipulations of IGFBP-3 expression. (**B**,**C**) UMSCC38-shEV and UMSCC38-shBP3 cells transfected with empty (EV) or pCMV6-IGFBP3 (BP3) vectors were treated with cycloheximide (CHX; 100 μg/mL) for the indicated time intervals and subjected to Western blot (WB) analysis on vimentin expression. (**C**) The relative expression level of vimentin was determined by densitometric analysis of vimentin expression at each time point normalized by the level of actin expression using the ImageJ software. (**D**,**E**) Paired UMSCC38-shEV and UMSCC38-shBP3 cells (**D**) and OSC19-EV and OSC19-BP3 cells (**E**) were treated with MG132 (10 µM) for 6 h. Cell lysates were immunoprecipitated with the anti-vimentin antibody, followed by WB analysis with the anti-ubiquitin antibody. Whole cell lysates (WCL) were also included for WB analysis. (**F**) UMSCC38-shEV (left) and UMSCC-shBP3 transfected with control (EV) or IGFBP-3 expression vector (right) were immunoprecipitated with preimmune serum (IgG) or anti-vimentin or anti-IGFBP-3 antibodies. The interaction between IGFBP-3 and vimentin was determined by WB analysis. (**G**) OSC19-Luc cells were transiently transfected with empty (EV) or pCMV6-IGFBP3 (BP3) vectors. Cell lysates were immunoprecipitated with the anti-vimentin antibody. Vimentin immunoprecipitates or whole-cell lysates (WCL) were subjected to WB analysis to determine the interaction between IGFBP-3 and vimentin. (**H**) Pull-down assays to determine the interaction between IGFBP-3 and vimentin. Ni-NTA agarose-bound recombinant His-IGFBP-3 (top) or glutathione (GSH)-agarose-bound recombinant GST-vimentin (bottom) proteins were incubated with UMSCC38 cell lysates. Ni-NTA agarose (top) or GSH-agarose-bound GST (bottom) were used to ensure specific interaction. (**I**) UMSCC38-shBP3 cells were transiently transfected with pCMV6 (EV) or pCMV6-IGFBP3 (BP3) and treated with MG132 (10 μM) for 6 h. The co-localization between IGFBP-3 and vimentin was determined by IF analysis. Scale bars: 20 µm. The bar represents mean ± SD. * *p* < 0.05 and ** *p* < 0.01, as determined by one-way ANOVA followed by Dunnett’s post-hoc test (**C**). Vim: vimentin; BP3: IGFBP-3; OSC19: OSC19-Luc; Ub: ubiquitin; NS: nonspecific band.

We investigated the mechanism underlying IGFBP-3-mediated downregulation of vimentin protein levels. We found that *VIM* mRNA expression remained unchanged in the HNSCC and NSCLC cell lines after the manipulation of IGFBP-3 expression (Figure 4A). Therefore, IGFBP-3 appears to regulate vimentin expression through a post-transcriptional mechanism.

We then determined the effect of IGFBP-3 on the half-life of vimentin protein in UMSCC38 cells after blocking de novo protein synthesis by cycloheximide (CHX) treatment [44]. Intriguingly, the half-life of vimentin protein was significantly longer in UMSCC38-shBP3 (UM38-shBP3) cells than in their corresponding control (UM38-shEV) cells, while overexpression of IGFBP-3 in UM38-shBP3 cells markedly destabilized vimentin protein (Figure 4B,C). These findings indicate IGFBP-3-mediated regulation of vimentin protein stability. Protein stability is frequently regulated by proteolysis via the ubiquitin-proteasome pathway [45] and the ubiquitin modification of vimentin was suggested in a previous report [46]. Hence, we investigated whether the ubiquitin-proteasome pathway was involved in the effect of IGFBP-3 on vimentin protein stability. As shown in Figure 4D, UM38-shEV cells exhibited polyubiquitination of vimentin after treatment with MG132, whereas vimentin ubiquitination was markedly ablated in UM38-shBP3 cells. Conversely, IGFBP-3 overexpression enhanced vimentin ubiquitination in OSC19-Luc (OSC19) cells (Figure 4E). We then assessed the interaction between IGFBP-3 and vimentin. Immunoprecipitation (IP) assays using MG132-treated UM38-shEV cells revealed a clear association of vimentin with IGFBP-3 (Figure 4F, left). UM38-shBP3 (Figure 4F, right) and OSC19-Luc cells (Figure 4G) with low endogenous IGFBP-3 levels showed undetectable levels of IGFBP-3-vimentin association, while ectopic IGFBP-3 expression showed a clear association between IGFBP-3 and vimentin. We next generated a bacterial expression vector carrying His-tagged IGFBP-3 or GST-tagged vimentin. Pull-down assays using IGFBP-3 and vimentin recombinant bacterial proteins and UMSCC38 cell lysates revealed an interaction between IGFBP-3 and vimentin (Figure 4H). IF analysis further showed co-localization of vimentin and IGFBP-3 in the cells, wherein proteasome activity was suppressed by treatment with MG132, confirming the vimentin-IGFBP-3 interaction (Figure 4I). These results suggest that IGFBP-3 interacts with vimentin and negatively regulates its stability through proteasome-mediated degradation.

### 2.5. The C-Terminal Domain of IGFBP-3 Directly Binds to the Head Domain of Vimentin

**Figure 5:**
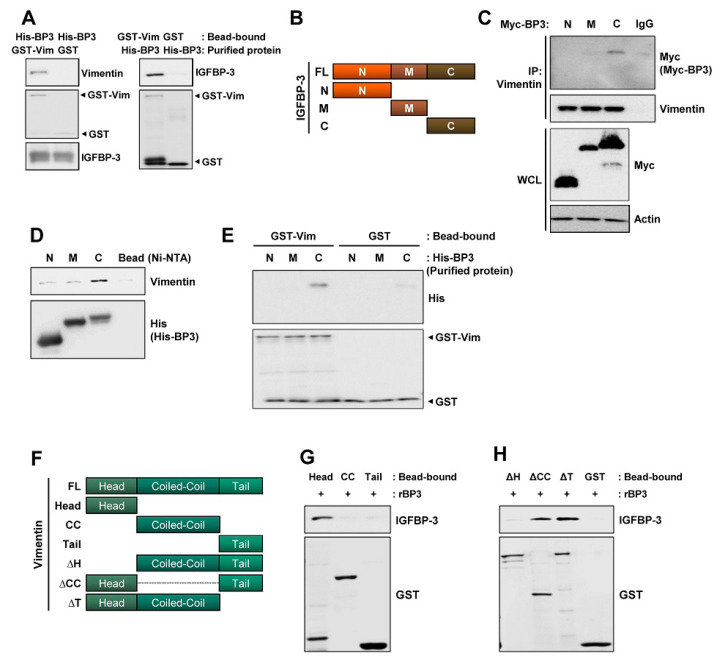
C-terminal domain of IGFBP-3 and the head domain of vimentin are critical for the binding of IGFBP-3 to vimentin. (**A**) Pull-down assays to determine the interaction between IGFBP-3 and vimentin. Purified and bead-bound proteins in each pull-down set were indicated. (**B**) Schematic diagram illustrating full-length and domain constructs (N: N-terminal domain; M: middle domain; C: C-terminal domain) of the IGFBP-3 protein. (**C**) HEK293T cells were transfected with Myc-tagged IGFBP-3 domain constructs for 48 h. Interaction between vimentin and C domain of IGFBP-3 was determined by immunoprecipitation with the anti-vimentin antibody and subsequent Western blot (WB) analysis. *Bottom*. Expression of each IGFBP-3 domain was determined by WB analysis. (**D**) Pull-down assay for the interaction between N-, M-, C-terminal domain of IGFBP-3 and endogenous vimentin protein. The Ni-NTA agarose-bound His-tagged IGFBP-3 domains (N, M, and C) were incubated with UMSCC38 cell lysates. The interaction between each IGFBP-3 domain and vimentin was determined by WB analysis. (**E**) Direct interaction between glutathione-agarose-bound GST-vimentin (GST-Vim) and C-terminal domain of IGFBP-3 was determined by a pull-down assay. (**F**) Schematic diagram illustrating full-length (FL) and domain constructs [Head: head domain; CC: coiled-coil domain; Tail: tail domain; ΔH: head domain deletion mutant; ΔCC: coiled-coil domain deletion mutant; ΔT: tail domain deletion mutant] of the vimentin protein. (**G**) Direct interaction between recombinant IGFBP-3 (rBP3) and the head domain of vimentin was determined by a pull-down assay. *Bottom*. Expressions of each vimentin domain were determined by WB analysis using the anti-GST antibody. (**H**) Disruption of the direct interaction with IGFBP-3 by deletion of the head domain (ΔH domain) of vimentin was determined by a pull-down assay. *Bottom*. Expressions of each deletion mutants were determined by WB analysis using the anti-GST antibody. Vim: vimentin; BP3: IGFBP-3.

IGFBP-3 interacts with proteins on the cell surface (e.g., LRP1 and TMEM219 [47]) or extracellular matrices [48]. Hence, we assessed whether IGFBP-3 had direct physical interaction with vimentin and found a direct interaction between IGFBP-3 and vimentin (Figure 5A). To define the crucial domains of IGFBP-3 and vimentin for their interaction, we established expression vectors carrying Myc-tagged N-terminal (N), middle (M), and C-terminal (C) domains of human IGFBP-3 [49] (Figure 5B) and transfected HEK293T cells with these vectors. IP assays using anti-vimentin antibodies and HEK293T cell lysates showed that vimentin specifically co-immunoprecipitated with the C domain of IGFBP-3 (Figure 5C). We further generated bacterial expression vectors carrying the three deletion mutants with the N-, M-, or C-terminal residues of IGFBP-3. A pull-down assay also showed a strong interaction between the C-terminal residue of recombinant IGFBP-3 protein and endogenous vimentin protein (Figure 5D). We further confirmed a direct interaction between the C-terminal IGFBP-3 and the GST-tagged recombinant full-length vimentin protein (Figure 5E).

Human vimentin protein is composed of a typical tripartite domain structure with a central α-helical coiled-coil (CC) rod domain flanked by non-α-helical amino- and carboxy-terminal (head and tail) domains [50,51]. To define the vimentin domain that is critical for IGFBP-3 binding, we generated GST-tagged head, coiled-coil (CC), and tail domains of recombinant vimentin proteins, and performed an in vitro protein binding assay with recombinant IGFBP-3 protein (Figure 5F). The head domain of vimentin was able to interact with the recombinant IGFBP-3 protein (Figure 5G). The interaction between the C domain of IGFBP-3 and the head domain of vimentin was further confirmed by the in vitro binding assay using domain deletion mutants of the recombinant vimentin protein. Deletion of either the CC or the tail domain of vimentin did not affect its interaction with IGFBP-3, whereas the deletion of the head domain markedly abolished this interaction (Figure 5H). These results indicate that the C-terminal domain of IGFBP-3 and the head domain of vimentin are responsible for the direct interaction between the two proteins.

### 2.6. IGFBP-3 Induces Proteasome-Mediated Degradation of Vimentin by Mediating the Complex Formation Between Vimentin and the F-Box E3 Ubiquitin Ligase FBXL14

**Figure 6:**
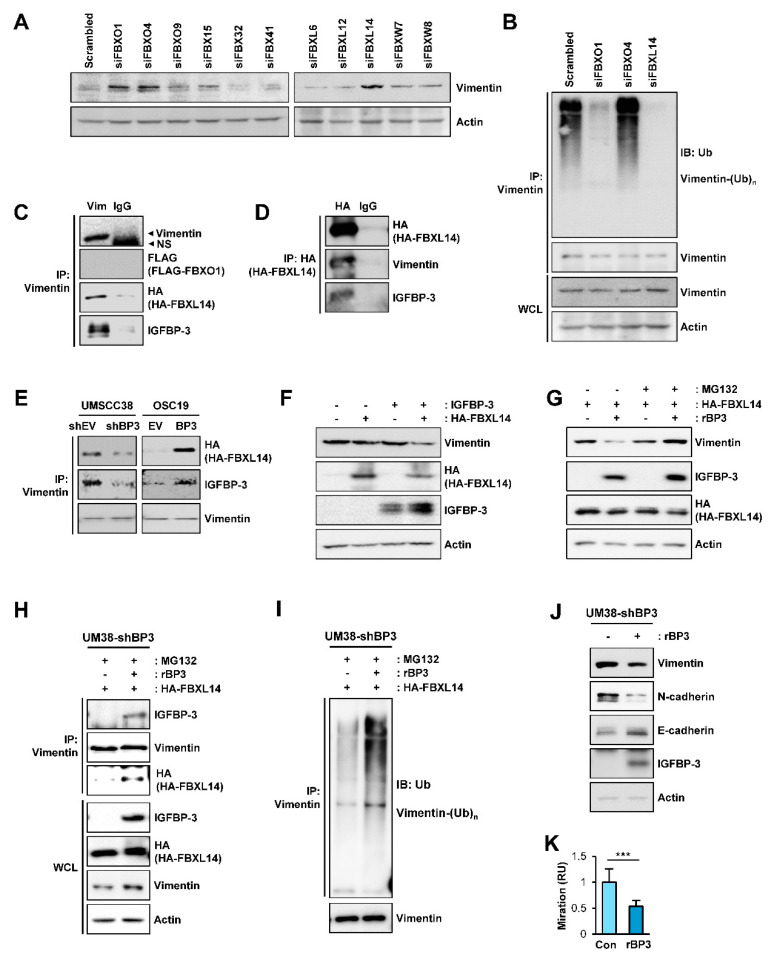
Association with FBXL14 is involved in the IGFBP-3-mediated proteasomal degradation of the vimentin protein. (**A**) UMSCC38 cells were transiently transfected with various siRNAs targeting F-box proteins. Expression of vimentin was assessed by western blot (WB) analysis. (**B**) UMSCC38 cells were transiently transfected with the indicated siRNA for 48 h, treated with MG132 for 6 h, and then followed by immunoprecipitation with the anti-vimentin antibody. Polyubiquitinated vimentin was detected by WB analysis. (**C**,**D**) HEK293T cells were transiently transfected with Flag-tagged FBXO1(**C**) or HA-tagged FBXL14 (**C**,**D**) expression vectors and then treated with MG132 (10 µM) for 6 h. The interaction among vimentin, IGFBP-3, and FBXL14 was determined by immunoprecipitation (IP) of HEK293T lysates with anti-vimentin (**C**) or anti-HA (**D**) antibodies, followed by WB analyses on the indicated proteins. (**C**) No overt interaction among vimentin, IGFBP-3, and FBXO1 was determined by immunoprecipitation with the anti-vimentin antibody, followed by WB analysis. (**E**) Cells were transfected with a HA-FBXL14 expression vector and then treated with MG132 (10 µM) for 6 h. Vimentin association with FBXL14, IGFBP-3 was determined by IP of indicated cell lysates with the anti-vimentin antibody, followed by WB analysis on the indicated proteins. (**F**) WB analysis on the indicated proteins in HEK293T cells in which empty vectors (-), pCMV6-IGFBP3, a HA-FBXL14 expression vector, or their combination were transiently transfected. (**G**–**I**) UM38-shBP3 cells were transiently transfected with HA-FBXL14 expression vector, treated with vehicle or rBP3 (5 µg/mL) for 48 h, and then untreated or treated with with MG132 (10 μM) for 6 h. WB analyses for the indicated protein expressions (**G**) and IP analyses for the interaction among vimentin, rBP3 and FBXL14 (**H**) and induction of the polyubiquitination of vimentin (**I**) are shown. (**J**) WB analysis showing regulation of EMT-associated markers by treatment with rBP3. (**K**) The Transwell migration assay for the regulation of UM38-shBP3 cell migration by treatment with rBP3. The bar represents mean ± SD. *** *p* < 0.001, as determined by two-tailed Student’s *t*-test. Vim: vimentin; BP3: IGFBP-3; Ub: ubiquitin; NS: nonspecific band.

Given the IGFBP-3-induced polyubiquitination of vimentin, we assessed the E3 ligases involved in the IGFBP-3-mediated polyubiquitination of vimentin. In the ubiquitin-proteasome pathway, the SKP-Cullin1-F-box (SCF). which is composed of the S phase kinase adapter protein, scaffold protein Cullin1, and F-box proteins, is the largest family of ubiquitin ligase complexes [52]. In SCF complexes, F-box E3 ubiquitin ligases recognize various biological and oncogenic substrates for ubiquitination and protein degradation [52]. To determine potential ubiquitin ligases mediating the degradation of vimentin protein by IGFBP-3, we screened an siRNA library comprising of siRNAs targeting 11 F-box E3 ligases, including FBXO1, FBXO4, FBXO9, FBX15, FBX32, FBX41, FBXL6, FBXL12, FBXL14, FBXW7, and FBXW8. As shown in Figure 6A, siRNA-mediated silencing of FBXO1, FBXO4, and FBXL14 upregulated vimentin expression, which was consistent with previous reports demonstrating the association of these F-box proteins with the regulation of several mesenchymal markers, including vimentin [53,54,55,56]. We next determined whether genomic ablation of the selected F-box protein affects the polyubiquitination of vimentin in UMSCC38 cells. We observed that the polyubiquitination of vimentin was markedly absent in cells in which siRNA-mediated FBXO1 or FBXL14 ablation was achieved (Figure 6B).

We identified the E3-ligase that mediates IGFBP-3-induced vimentin degradation by performing IP analysis. IP analyses using HEK293T cells that were transiently cotransfected with empty vectors or expression vectors for vimentin, IGFBP-3, FBXO1, or FBXL14 prior to MG132 treatment shown that vimentin immunoprecipitates were clearly associated with IGFBP-3 and FBXL14, but not with FBXO1 (Figure 6C). Consistently, HA-tagged FBXL14 immunoprecipitates were associated with vimentin and IGFBP-3 (Figure 6D). In addition, the interaction between vimentin and FBXL14 was evident in the presence of IGFBP-3 (UMSCC38-shEV and OSC19-BP3 cells), and such association was markedly abolished in UMSCC38-shBP3 and OSC19-EV cells (Figure 6E). We further observed markedly reduced vimentin expression in HEK293T cells with ectopic overexpression of both IGFBP-3 and FBXL14, but not in those of IGFBP-3 or FBXL14 alone (Figure 6F). These results suggest that IGFBP-3 mediated destabilization of vimentin protein by inducing association with vimentin and the E3 ubiquitin ligase FBXL14.

### 2.7. IGFBP-3 Protein Is Internalized into the Cells and Mediates the Complex Formation between Vimentin and FBXL14, Leading to Proteasome-Mediated Degradation of Vimentin

Because the IGFBP-3 expression vector used in this study did not contain the sequence for signal peptide and vimentin has been known to localize in the cytosol or nucleus [57], the interaction among IGFBP-3, vimentin, and FBXL14 appeared to be an intracellular event. Considering the nature of IGFBP-3 as a secretory protein and cellular uptake of extracellular IGFBP-3 via clathrin-mediated endocytosis, caveolin-dependent pathway, and fluid-phase endocytosis [58,59], we postulated that extracellular IGFBP-3 protein internalizes and regulates vimentin expression and EMT-associated phenotypes. In support of the notion, a recent study showed cellular uptake of extracellular recombinant IGFBP3 (rBP3) protein, leading to interaction between GRP78 and IGFBP-3 [60]. To confirm the hypothesis, vehicle or rBP3 protein in full-length [rBP3 (FL)] was added to UM38-shBP3 cells, wherein FBXL14 expression was induced by transiently transfection. These cells were remained untreated or treated with MG132. After an extensive washout of the treated cells to remove rBP3, we performed western blot analyses of the whole cell lysates (WCLs). The rBP3-treated cells showed clear IGFBP-3 protein along with an obvious decrease in vimentin expression. Moreover, the rBP3-induced inuced decrease in vimentin expression was abrogated by the treatment with MG132 (Figure 6G). Immunoprecipitation analysis further revealed an association among IGFBP-3, vimentin, and FBL14 in the rBP3-treated cells (Figure 6H) and polyubiquitination of vimentin (Figure 6I). We further confirmed that treatment with rBP3 downregulated vimentin and N-cadherin expression and elevated E-cadherin expression in UM38-shBP3 cells (Figure 6J). In addition, treatment with rBP3 significantly suppressed migration of UM38-shBP3 cells, a typical EMT-associated phenotype [30] (Figure 6K). Taken together, these results suggested that IGFBP-3 internalizes and makes a complex formation with vimentin and FBXL14, thereby causing polyubiquitination and proteasomal degradation of vimentin and suppressing EMT-associated metastatic phenotypes of cancer cells.

## 3. Discussion

**Figure 7:**
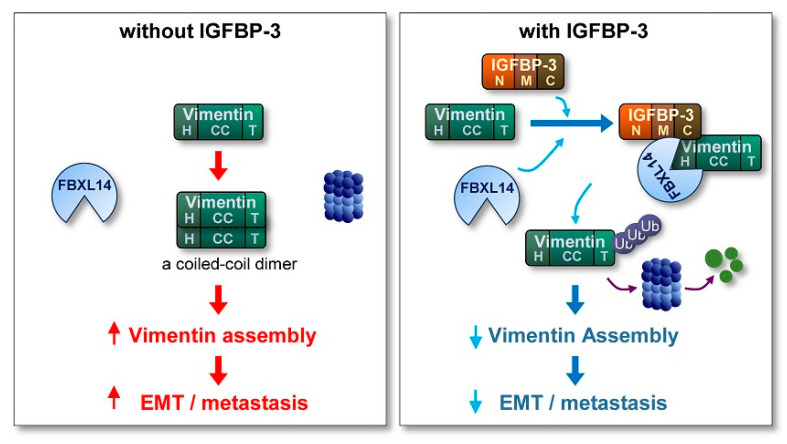
Schematic model of the mechanism underlying the antimetastatic effect of IGFBP-3. In the absence of IGFBP-3, vimentin protein, which is composed of a central α-helical coiled-coil (CC) domain capped on each side by amino (head; H) and carboxyl (tail; T) domains, forms a coiled-coil dimer, the basic subunit of vimentin assembly, eventually stimulating the EMT program and metastasis of cancer cells. In the presence of IGFBP-3, the vimentin head domain makes a direct interaction with the IGFBP-3 C-terminal domain, resulting in the recruitment of the ubiquitin ligase FBXL14 and proteasomal degradation of vimentin. Consequently, assembled vimentin proteins required for the formation of intermediate filament is reduced, eventually suppressing the EMT program and metastasis.

Metastasis, the spread of cancer cells from primary tumors to surrounding tissues or distant organs [61,62,63], is the main cause of cancer-related death [61]. However, because of the biological complexity of metastasis, no therapeutic regimens that block metastatic tumors are clinically available. Hence, understanding the biology of metastatic cancer cells and development of efficacious anticancer strategies targeting key molecules that support migration and invasion of cancer cells are logical steps to improve patient outcomes. Here, we show the ability of IGFBP-3 to inhibit the metastatic potential of NSCLC and HNSCC. Our results specifically emphasize an IGF-independent action of IGFBP-3 in regulating EMT phenotypes and metastatic activities of NSCLC and HNSCC. In our model, IGFBP-3 destabilizes the mesenchymal marker vimentin by mediating the complex formation between vimentin and the E3 ubiquitin ligase FBXL14, causing proteasome-mediated degradation of vimentin (Figure 7). Our results specifically emphasize a unique mechanism by which the C-terminal domain of IGFBP-3 directly binds to the head domain of vimentin. Collectively, our results highlight that IGFBP-3 acts as an inhibitor of cancer cell metastasis.

A great deal of cancer research has focused on understanding the molecular mechanisms underlying cancer cell metastasis and discovering strategies to inhibit cancer cell metastasis. A small population of primary tumor cells is known to metastasize to distant organs, undergoing multiple steps, including detachment from primary tumors, degradation of the surrounding extracellular matrix, intravasation, evasion of immunity, extravasation, invasion to distant organs, and metastatic colonization [63]. Several factors, intrinsically derived from cancer cells or extrinsically derived through communication with surrounding stromal cells, are involved in this cascade [61,63]. Given the antiproliferative, proapoptotic, antiangiogenic, and anti-adhesion effects of IGFBP-3 in a variety of cancer cells [20,21,64], we postulated the antimetastatic activity of IGFBP-3 in NSCLC and HNSCC. In support of this notion, IGFBP-3 has shown potential as a metastasis suppressor in prostate, lung, and ovarian cancer [22,23]. In line with these previous findings, modulation of IGFBP-3 expression apparently influenced metastatic activities of HNSCC and NSCLC cells in various experimental models: (1) IGFBP-3 expression modulated EMT phenotypes and migratory and invasive capacities of HNSCC and NSCLC cells; (2) HNSCC-induced metastatic tumor formation in the lungs was potentiated by the ablation of *Igfbp3* expression in the 4-NQO-induced HNSCC tumorigenesis model; (3) silencing IGFBP-3 expression elevated the capacity of HNSCC in orthotopic tumors to locally invade into the lymph node mouse model; and (4) loss of IGFBP-3 enhanced the metastatic potential of NSCLC cells.

We investigated the IGFBP-3 downstream targets involved in metastatic activities. IGFBP-3 has been shown to regulate tumor growth and angiogenesis by modulating IGF bioavailability [20]. IGFBP-3 also has IGF-independent antitumor activities through interaction with cell surfaces or intracellular proteins [65], regulation of Egr1-mediated transcriptional events [20], and downregulation of integrin signaling [21]. Moreover, our previous studies revealed that reduced IGFBP-3 expression is a frequent event in the early stage of NSCLC, which correlated with the disease-specific survival probability of stage I NSCLC patients (*p* = 0.02) [66]. Stage I NSCLC patients who lost IGFBP-3 expression due to the hypermethylation at the CpG sites of IGFBP-3 promoter exhibited statistically lower overall survival (*p* = 0.022), disease-specific survival probability *p* = 0.006), and disease-free survival probability *p* = 0.007) 5 years after diagnosis [67]. The prognostic role of IGFBP-3 has been previously reported in head and neck cancer [68]. However, it is largely unknown whether and how IGFBP-3 regulates the EMT program. Strikingly, our findings show the IGF-independent capacity of IGFBP-3 to suppress vimentin expression, a key molecule regulating the EMT process [9,69]. Acquisition of EMT phenotypes is a typical intrinsic change in cancer cells in the initial stage of metastasis. Ordinarily, the EMT program is critical for several developmental and pathophysiological processes such as embryogenesis, organ development, wound healing, tissue regeneration, and organ fibrosis [70]. Cancer cells acquire EMT through undefined genetic or epigenetic changes favoring clonal outgrowth, and utilize this process to invade and metastasize locally or systemically [70]. Studies have shown that vimentin induces mesenchymal shaping of epithelial cells and promotes the adhesion and motility of various types of cells by patterning microtubules, restricting actin flow, regulating traction stresses, supporting lateral cell-to-cell contacts, and upregulating contact-dependent cell stiffening [9,71]. Vimentin contributes to cancer cell metastasis by maintaining heterotypic tumor cells during the collective invasion [72]. In addition to these regulatory effects on the cytoskeleton, vimentin regulates the signaling pathways associated with cell adhesion, EMT, and cell polarization by forming the VAV2-vimentin-FAK complex [73], acting as a scaffold for interaction between Slug and ERK [16], and protecting Scribble from proteasomal degradation [74]. In line with these previous reports, vimentin showed the capacity to stimulate migration of HNSCC and NSCLC cells. Our findings show the IGF-independent capacity of IGFBP-3 to directly bind to and destabilize vimentin protein via ubiquitin-mediated proteasomal degradation. A previous study showed that targeting vimentin using the vimentin binding molecule FiVe1 promoted its degradation via the ubiquitin-proteasome pathway, leading to rearrangement of cancer cell morphology to a more epithelium-like state [75]. Thus, collapse of vimentin architecture and subsequent modification of its assembly/disassembly may be the mechanism underlying IGFBP-3-mediated regulation of cancer cell migration and invasion.

Notably, the C-terminal domain of IGFBP-3 and the head domain of vimentin appeared to be responsible for the interaction between the two proteins. The head domain of vimentin was found to contain several sites for phosphorylation and glycosylation, which play an essential role in the assembly of vimentin [76]. Akt was found to phosphorylate the head domain of vimentin at Ser39, enhancing the ability of vimentin to promote motility and invasion of cells [69]. The C-terminal domain of IGFBP-3 contains several functional motifs, including the nuclear localization sequence and the binding sites for heparin and the acid-labile subunit (ALS) [77]. Therefore, it is plausible to say that IGFBP-3 might regulate the motility of cancer cells by interfering with the interaction between vimentin and Akt.

Although IGFBP-3 is a secretary protein. IGFBP-3 is known to intracellularly transported via the transferrin-receptor mediated internalization, caveolae-mediated endocytosis, or fluid-phase endocytosis and can interact with several intracellular proteins localized in the cytoplasm and nucleus [58,59]. In addition, vimentin is an intermediate filament protein localized in the cytoplasm and also found to localize in the nucleus and play a role in regulating nuclear architecture and perinuclear stiffness [57]. Our results suggest that exogenously added IGFBP-3 can internalize into the cells, forms a complex with vimentin and FBXL14, and induces polyubiquitination and proteasomal degradation of vimentin, indicating that vimentin may intracellularly interact with IGFBP-3.

Our present study reveals a novel mechanism of vimentin regulation, in which IGFBP-3 mediates complex formation between vimentin and ubiquitin ligase FBXL14, resulting in proteasomal degradation of vimentin. It was also noted that ectopic expression of FBXL14 reduced vimentin level in breast cancer cells [53]. In addition to vimentin, FBXL14 regulates the stability of EMT transcription factors such as Snail and Twist by inducing their proteasomal degradation [55,56]. Another EMT transcription factor, slug, is also degraded by an SCF ubiquitin ligase Ppa, a homologue of FBXL14 in *X. laevis* embryos [78]. Therefore, FBXL14 appears to be a common regulator of the EMT program and targeting FBXL14 may control the EMT-mediated metastatic process in cancer cells. In line with this notion, the role of FBXL14 in the regulation of metastasis was evidenced by previous studies [53,55]. Although further studies are required to uncover the detailed mechanism through which FBXL14 deregulates the stability of vimentin, IGFBP-3 appears to regulate metastasis by mediating the interaction between vimentin and E3 ligase, leading to disruption of vimentin stability, causing its disassembly.

In conclusion, our results reveal, for the first time, that vimentin is a cellular target for IGFBP-3-mediated suppression of metastasis. Our results also explain the IGF-independent inhibitory actions of IGFBP-3 in migration and invasion of aerodigestive tract cancer cells. IGFBP-3 functions as a master enforcer of vimentin destabilization through its cooperation with FBXL14. A loss of IGFBP-3 expression due to the methylation or polymorphisms of the IGFBP-3 promoter was frequently detected in HNSCC and NSCLC [67,79]. These findings highlight the vimentin-FBXL14 complex as a potential target for the development of therapeutic strategies targeting the EMT-induced metastatic process. Further translational research is required to critically evaluate the potential of IGFBP-3, especially its C-terminal domain, as a novel strategy to control the metastatic progression of aerodigestive tract cancers.

## 4. Materials and Methods

### 4.1. Reagents

Antibodies against E-cadherin, N-cadherin, and Matrigel basement matrix were purchased from BD Biosciences (San Jose, CA, USA). Antibodies against IGFBP-3, vimentin, myc, glutathione S-transferase (GST), His-probe, HA-probe, OctA-probe, IGF-1R, ubiquitin, and actin were purchased from Santa Cruz Biotechnology (Dallas, TX, USA). Antibodies against E-cadherin, N-cadherin, and tubulin were purchased from Cell Signaling Technology (Danvers, MA, USA). Horseradish peroxidase (HRP)-conjugated secondary antibodies were purchased from GeneTex (Irvine, CA, USA). Fluorochrome (Alexa Fluor 488, Alexa Fluor 546, or Alexa Fluor 594)-conjugated secondary antibodies were purchased from Thermo Fisher Scientific (Waltham, MA, USA). Human recombinant IGFBP-3 was purchased from R&D Systems (Minneapolis, MN, USA). Human recombinant IGFBP-3 protein used or evaluation of cellular uptake of extracellular IGFBP-3 was kindly provided by Insmed Inc. (Glen Allen, VA, USA) [22]. 3-(4,5-dimethylthiazol-2-yl)-2,5-diphenyltetrazolium bromide (MTT) was purchased from MP Biomedicals (Santa Ana, CA, USA). G418 was purchased from Enzo Life Sciences (Farmingdale, NY, USA). Crystal violet, a mouse monoclonal anti-vimentin antibody, and additional chemicals unless otherwise indicated were purchased from Sigma-Aldrich (St. Louis, MO, USA).

### 4.2. Cell Culture

Human HNSCC cell lines (UMSCC1, UMSCC4, UMSCC14A, UMSCC38, and OSC19-Luc) were kindly provided by Dr. Jeffrey N. Myers (MD Anderson Cancer Center, Houston, TX, USA). The human NSCLC cell line H226B was kindly provided by Dr. John V. Heymach (MD Anderson Cancer Center). The human HNSCC cell line FADU, human NSCLC cell line H1299, and the human embryonic kidney 293T cell lines (HEK293T) were purchased from the American Type Culture Collection (ATCC; Manassas, VA, USA). Cells were cultured in Dulbecco’s modified Eagle’s medium (DMEM; for UMSCC1, UMSCC4, UMSCC14A, FADU, OSC19-Luc, and HEK293T cell lines), Dulbecco’s modified Eagle’s medium/nutrient mixture F-12 (DMEM/F12; for UMSCC38 cells), or RPMI 1640 medium (for NSCLC cell lines), supplemented with 10% fetal bovine serum (FBS) and antibiotics (all from WelGENE, Kyeongsan-si, Republic of Korea), and maintained at 37 °C with 5% CO_2_ in a humidified atmosphere. We used cells that were passaged for fewer than 20 times after resuscitation.

### 4.3. Plasmids, siRNAs, and shRNAs

The plasmid constructs for IGFBP-3 expression are described in our previous reports [20,21]. The IGFBP-3 expression vector used in this study does not contain the signal peptide sequence. The plasmid constructs for the His-tagged bacterial full-length IGFBP-3 protein and its domain constructs were generated by cloning them into the pET32a vector. Cloning details are available on request. The scrambled small interfering RNA (siRNA) control was purchased from Dharmacon (Thermo Fisher Scientific). The pEGFP-C1-vimentin expression vector containing full-length (1-467 aa) vimentin was kindly provided by Dr. Dale D. Tang (Albany Medical College, Albany, NY, USA) [80]. mCherry-Cyclin F (FBXO1) was a gift from Michele Pagano (Addgene plasmid #32975; http://n2t.net/addgene:32975, accessed on 15 February 2021; RRID: Addgene_32975) (Addgene, Watertown, MA, USA) [81]. shRNAs for silencing *IGFBP3*, *IGF1R,* and pLKO.1-puro empty vector control were purchased from Open Biosystems (Thermo Fisher Scientific) and Sigma-Aldrich.

For GST-tagged bacterial protein expression, pEGFP-C1-vimentin was subcloned into the BamHI/EcoRI site of pGEX-4T-2 (GE Healthcare Life Sciences, Chicago, IL, USA). Head (1–101 aa), Coiled coil (102–410 aa), Tail (411–467 aa), ∆H (102–467 aa), ∆C (1–101 linked with 411–467 aa), and ∆T (1-410 aa) vimentin mutants were generated using the template (pGEX-4T-2-FL-vimentin) and the following primers: Head: forward 5′-TATGGATCCATGTCCACCAGGTCC-3′, reverse 5′-CCGGAATTCCTAGGGTGCG GGTGTTCTTGAACTC-3′; Coiled coil: forward 5′-CGCGGATCCAACG AGAAGGTGGAGCTGCAG-3′, reverse 5′-CCGGAATTCCTA-CCTGCTCTCCTCGCCTTCCAG-3′; Tail: forward 5′-CGCGGATCCATTTCTCTGCCTCTTCCAAAC-3′, reverse 5′-CCGGAATTCTTATTCAAGGTC-ATCGTGATGCTG-3′; ∆H: forward 5′-CGCGG ATCCAACGAGAAGGTGGAGCTGCAG-3′, reverse 5′-CCGGAATTCTTATTCA AGGTCATCGTGATGCTG-3′; ∆C: forward1 5′- /TATGGATCCATGTCCACCAGGTCC-3′, reverse1 5′-AAGAGGCAGAGAAATGGTGCGGGTGTTCTTGAA-3′, forward2 5′-AAG AACACCCGCACCATTTCTCTGCCTCTTCCA-3′, reverse2 5′-CCGGAATTCTTA-TTC AAGGTCATCGTGATGCTG-3′; ∆T: forward 5′-TATGGATCCAT GTCCACCAGGTCC-3′, reverse 5′-CCGGAATTCCTA-CCTGCTCTCCTCGCCTTCCAG-3′. For ∆C mutant vimentin, PCR products of head domain (forward 1 and reverse 1 primers) fragments and tail domain (forward 2 and reverse 2 primers) fragments were ligated and subcloned into pGEX-4T-2 using BamHI/EcoRI restriction sites.

### 4.4. Transfection

For transient transfection, we used expressing vectors or siRNAs using JetPRIME (Polyplus transfection, Illkirch, France), Fugene 6 (Promega, Madison, WI, USA) or Lipofectamine 2000 (Thermo Fisher Scientific) according to the manufacturer’s instructions. To establish which stable cell lines knocked down *IGFBP3* or *IGF1R* expression, lentiviral particles containing either the empty vector (pLKO.1; shEV), *IGFBP3,* or *IGF1R* shRNAs were produced by transfection of HEK293T cells with shRNAs, lentiviral packaging plasmid (pHR8.2deltaR), and the envelope plasmid (pCMV-VSV-G) using Fugene 6. The supernatants were collected 24 h and 48 h after transfection and then filtered through a 0.22 µm syringe filter. H226B, UMSCC1, UMSCC38, and H1299 cells were transduced with lentiviral supernatants containing shEV, shIGFBP3 (for H226B and UMSCC1), or shIGF1R (UMSCC38 and H1299) in the presence of 8 µg/mL polybrene for 24 h, and then the culture medium was replaced with medium containing 1—2 µg/mL puromycin for selection. Stable transfectants were selected for 3 weeks. Generations of UMSCC38 cells that were stably transfected with control or IGFBP3 shRNAs were described in our previous report [21]. To establish stable cell lines overexpressing IGFBP-3, OSC19-Luc, and H1299, cells were transfected with pCMV6 (EV) or pCMV6-IGFBP-3 (BP3) vectors using JetPRIME for 48 h. Transfected cells were selected using G418.

### 4.5. Cell Proliferation Assay

Cells were seeded in 24-well plates at a density of 5 × 10^4^ cells/well. The number of cells at different time points was determined by cell counting using a hemocytometer.

### 4.6. Scratch Assay

Cells were plated into 6-well plates. Confluent cells were scraped in a straight line with a sterile yellow tip to create a scratch and then washed with media to remove cell debris. Cells were photographed immediately after creation of a scratch and at different time points up to 24 h using the EVOS FL Cell Imaging System (Thermo Fisher Scientific). The distance between the sides of a scratch was measured using ImageJ software (National Institutes of Health, Bethesda, MD, USA) [82].

### 4.7. Migration and Invasion Assays

For the migration assay, the outer membranes of the Transwell (Corning Inc., Cornng, NY, USA) were coated with 0.05% gelatin. For the invasion assay, the outer membranes of the Transwell were coated with gelatin, and the inner membranes were coated with Matrigel. Cells in serum-free medium were seeded into a 24-well plate Transwell inserts, which contained medium supplemented with 10% FBS at the bottom of the well. Cells were incubated from 12 h to 24 h, and the incubation time was dependent on the cell type. After incubation, the membranes were fixed with methanol for 5 min and then washed twice with phosphate-buffered saline (PBS). Cells were then stained with hematoxylin for 8 min and washed thrice with distilled water. Cells on the upper surface of the insert were removed using a cotton swab. The membranes were cut and mounted onto a glass slide. The migrated cells were examined using a microscope (Nikon Eclipse 80 i, Nikon Instrument Inc., Tokyo, Japan).

### 4.8. Immunofluorescence Staining

Cells were seeded on coverslips and fixed with methanol for 10 min. Subsequently, cells were blocked with blocking buffer (3% BSA in TBS containing 0.025% Triton X-100) and then incubated with primary antibodies [anti-IGFBP-3 (Santa Cruz), anti-vimentin (Santa Cruz), anti-E-cadherin (Cell Signaling), and anti-N-cadherin (Cell Signaling), all 1:400 dilution] overnight at 4 °C. After washing with blocking buffer, coverslips were incubated with fluorochrome-conjugated secondary antibodies (1:1000 dilution) diluted in blocking buffers. Coverslips were stained with 4′,6-diamino-2-phenylindole (DAPI) solution (50 ng/mL) and mounted onto a glass slide. The fluorescence signals were observed under a fluorescence microscope (Zeiss Axio Observer Z1, Carl Zeiss AG, Oberkochen, Germany).

### 4.9. Aldehyde Dehydrogenase Assay

The AldeRed Aldehyde dehydrogenase (ALDH) assay kit (Merck KGaA, Darmstadt, Germany) was used to identify cell populations with high ALDH enzymatic activity. Cells were suspended in AldeRed buffer and stained with AldeRed A588 for 40 min at 37 °C. Each group contained a blank sample (AldeRed A588 alone) and a positive control sample (AldeRed A588 plus N,N-diethylaminobenzaldehyde (DEAB)]. The fluorescence intensity was determined using flow cytometry (BD Biosciences, San Jose, CA, USA), and the sorting gates were established using a sample with DEAB treatment (negative control).

### 4.10. Sphere Formation Assay

Cells were seeded on ultra-low attachment 96-well plates (Corning, Inc.) in spheroid medium [DMEM-F12 supplemented with B27 supplements (Thermo Fisher Scientific), epidermal growth factor (EGF), basic fibroblast growth factor (bFGF), and antibiotics]. Cells were incubated at 37 °C and 5% CO_2_ for two weeks, or until spheres formed and reached volumes above 150 µm^3^.

### 4.11. Western Blot Analysis and Immunoprecipitation

For western blot analysis, cells were lysed in RIPA buffer [50 mM Tris-HCl (pH 8.0), 150 mM NaCl, 1% Triton X-100, 1% sodium deoxycholate, 0.1% SDS, 1 mM EDTA, and protease inhibitor cocktail (Roche Diagnostics GmbH, Mannheim, Germany)]. Cell lysates were collected by centrifugation at 13,000 rpm at 4 °C, and the protein concentration was determined using the BCA assay kit (Thermo Fisher Scientific). The proteins were resolved by sodium dodecyl sulphate-polyacrylamide gel electrophoresis (SDS-PAGE) and then transferred onto a polyvinylidene difluoride (PVDF) membrane. The membranes were incubated with blocking buffer [3% BSA in Tris-buffered saline containing 0.1% Tween-20 (TBST)] for 1 h at room temperature (RT). The membranes were further incubated with primary antibodies (1:1000 dilution) diluted in blocking buffer overnight at 4 °C. After washing with TBST, the membranes were incubated with HRP-conjugated secondary antibodies (1:5000 dilution) diluted in blocking buffer for 1—2 h at RT. After washing several times with TBST, the signals were visualized with the SuperSignal West Femto chemiluminescent substrate (Thermo Fisher Scientific) using the ImageQuant LAS 4000 imaging system (GE Healthcare).

For immunoprecipitation, cells were lysed in EBC lysis buffer [50 mM Tris-HCl (pH 8.0), 120 mM NaCl, 0.5% NP-40, and 1 mM EDTA) supplemented with a protease inhibitor cocktail (Roche). The reaction was formed by incubating 0.7–1 mg of the cell lysates with 1 µg of appropriate primary antibodies in 1.5 mL microtubes with rotation overnight at 4°C. Subsequently, the reaction was performed using Protein A agarose beads (Merck KGaA) for 2 h. The immune complex was washed three times with EBC lysis buffer, boiled with 6× SDS sample buffer, and then subjected to Western blot analysis as described above. The whole western blot figures can be found in the supplementary materials. 

### 4.12. Pull-Down Assay

The expression and purification of hexahistidine (6× His, His)-tagged recombinant IGFBP-3 or glutathione-S-transferase (GST)-tagged vimentin proteins were performed as described previously [83]. For a pull-down assay, the recombinant IGFBP-3 protein bound to the Ni-NTA agarose or the recombinant vimentin protein bound to the glutathione-agarose were incubated with 1 mg of cell lysates or purified proteins in TNE binding buffer [50 mM Tris-HCl (pH 8.0), 120 mM NaCl, and 0.1 M EDTA) with rotation for 2 h or overnight at 4 °C. The pull-down complexes were centrifuged and washed thrice with the lysis buffer before being resolved using SDS-PAGE and analyzed by Western blot analysis.

### 4.13. Real-Time Polymerase Chain Reaction

Total RNA was isolated using the phenol-chloroform extraction method and then transcribed into complementary DNA using TransScript first-strand cDNA synthesis kit (Transgen Biotech, Beijing, China) with oligo(dT)_18_ primer. For RT-PCR analysis, PCR was performed using 2× MyTaq Red Mix (Bioline, London, UK) and gene-specific primers (human *IGFBP3* forward, GAA GGG CGA CAC TGC TTT TTC; human *IGFBP3* reverse, CCA GCT CCA GGA AAT GCT AG; human *ACTB* forward, ACT ACC TCA TGA AGA TC; human *ACTB* reverse, GAT CCA CAT CTG CTG GAA). The following RT-PCR conditions were applied: an initial denaturation step at 94 °C for 5 min; 23–30 cycles of 94 °C for 30 s, 55–60 °C for 30 s, and 72 °C for 30 s; and a final elongation step at 72 °C for 5–7 min. The PCR products were separated by 2% agarose gel electrophoresis and visualized using a Gel Doc EZ System (Bio-Rad Laboratories, Hercules, CA, USA). Real-time polymerase chain reaction (PCR) was performed using a SYBR green reagent (Enzynomics, Daejeon, Republic of Korea) and gene-specific primers (human *VIM* forward, CGG CTG CGA GAG AAA TTG C; human *VIM* reverse, CCA CTT TCC GTT CAA GGT CAA G; human *ACTB* forward, GCG AGA AGA TGA CCC AGA TC; human *ACTB* reverse, GGA TAG CAC AGC CTG GAT AG) on an Applied Biosystems 7300 real-time PCR system (Applied Biosystems, Thermo Fisher Scientific). The following thermocycler conditions for real-time PCR were applied: pre-incubation at 95 °C for 15 min; 40 cycles of 95 °C for 10 sec, 60 °C for 20 sec, and 72 °C for 30 sec; and a final melting curve analysis to determine reaction specificity. Relative quantification of mRNA expression was performed using the comparative CT (cycle threshold) method as described previously [84].

### 4.14. Animal Experiments

All animal procedures were performed using protocols approved by the Seoul National University Institutional Animal Care and Use Committee. Mice had free access to standard mouse chow and water and were housed in temperature- and humidity-controlled facilities with a 12-h light/dark cycle. For the spontaneous HNSCC tumorigenesis model, 8-week-old male and female wild-type (WT; *Igfbp3^+/+^*) or *Igfbp3* KO mice (*Igfbp3^−/−^*, an FVB/N background) were administered 100 μg/mL 4-NQO via drinking water. After 16 weeks, primary tumorigenesis and metastatic tumor formation in the lungs were monitored by hematoxylin and eosin (H&E) staining of the tissues obtained from the euthanized mice.

For orthotopic xenograft experiments, UMSCC38 cells, stably transfected with empty vectors or *IGFBP3* shRNAs (UMSCC38-shEV or UMSCC38-shBP3 cells) [1 × 10^6^ cells, suspended in 50 μL of Matrigel diluted in ice-cold PBS (1:1)], were injected into the tongue of 6- to 8-week-old male and female non-obese diabetic/severe combined immunodeficiency (NOD/SCID) mice. After five weeks, the development of metastasis was monitored using IVIS SpectrumCT In Vivo Imaging System (PerkinElmer, Waltham, MA, USA) with a MMPSense 680 probe. Metastatic tumor formation in the cervical lymph nodes was also observed by H&E staining of the harvested tissues obtained from the euthanized mice.

For xenograft experiments, H226B cells, stably transfected with empty vectors or *IGFBP3* shRNAs (226B-shEV and 226B-shBP3 cells) [1 × 10^6^ cells, suspended in 100 μL of Matrigel diluted in ice-cold PBS (1:1)], were injected into the right flanks of 6- to 8-week-old male and female NOD/SCID mice. Because the growth of 226B-shBP3 tumor xenografts was slower than that of 226B-shEV tumors, the weights of the primary tumors and metastases in the lungs were monitored after the size of the 226B-shBP3 tumors increased until they were similar to the 226B-shEV tumors. Metastasis in the lungs was monitored by H&E staining of the tissues obtained from the euthanized mice.

Microscopic evaluations of the H&E-stained lung tissues were performed to measure the mean tumor number (N) and volume (V) in a blinded fashion. The tumor volume was calculated using the following formula: V (mm^3^) = (long diameter × short diameter^2^)/2, and the tumor load was calculated using the following formula: mean tumor number (N) × mean tumor volume (V). The number and sizes of tumors were calculated in five sections uniformly distributed throughout each lung.

### 4.15. Immunohistochemistry

Sections derived from formalin-fixed and paraffin-embedded (FFPE) tumor or lung tissues were deparaffinized by incubation overnight at 65 °C, followed by rehydration in sequential xylene and ethanol rinses. After treatment with hydrogen peroxide, the slides were washed with PBS and then incubated with 0.3% Triton X-100. After washing again with PBS, the sections were incubated with blocking solution (Dako Protein Block, Dako, Glostrup, Denmark) for 30 min at RT. The sections were further incubated with primary antibodies (IGFBP-3 and SOX2, diluted at 1:200) overnight at 4 °C. After washing multiple times with PBS, the sections were incubated with the corresponding biotinylated secondary antibodies (diluted at 1:500) for 1 h at RT. The sections were washed multiple times with PBS, treated with avidin-biotin complexes (Vector Laboratories, Burlingame, CA, USA), visualized using a diaminobenzidine detection reagent (Enzo Life Sciences), and then mounted with a mounting solution (Vector Laboratories).

### 4.16. In Silico Analysis

We analyzed the TCGA Firehose Legacy dataset of lung squamous carcinoma and two datasets available in the Gene Expression Omnibus (GEO) database (GSE3141 amd GSE8894) to determine the correlation between the IGFBP-3 mRNA expression and the mRNA expression of EMT markers (*VIM* and *CDH1*) in NSCLC. The normalized RNA-Seq by Expectation Maximization (RSEM) values of *IGFBP3*, *VIM*, and *CDH1* in each sample were manually downloaded from the cBioPortal website (http://www.cbioportal.org, accessed on 15 February 2021). For analysis of GEO datasets, raw data consisting of gene expression levels and clinical information for each patient sample were manually downloaded and analyzed. The following probes were used to obtain gene expression level of each gene: 212143_s_at for *IGFBP3*; 201130_s_at for CDH1; 1555938_x_at for VIM. The Spearman correlation coefficient and the significance of the correlation coefficient were determined using Graphpad Prism (version 9, GraphPad software, San Diego, CA, USA).

### 4.17. Statistics

Data are presented as the mean ± SD. All in vitro experiments were independently performed at least twice, and a representative result is shown. The values presented in the graphs were generated by multiple replicates in a representative experiment. Statistical significance was determined by a two-tailed Student’s *t*-test, Mann-Whitney test, or one-way analysis of variance (ANOVA) using GraphPad Prism (version 9, GraphPad software, San Diego, CA, USA). The Shapiro-Wilk test was performed to determine whether the in vitro or in vivo data follows a Gaussian distribution. Statistical significance was set at *p* < 0.05.

## 5. Conclusions

Our findings reveal the inhibitory effect of IGFBP-3 on cancer migration and metastasis by negatively regulating vimentin expression through ubiquitin-mediated proteasome degradation through the cooperation with the E3 ligase FBXL14 in aerodigestive tract cancer cells. This study highlights the potential of vimentin-FBXL14 complexes as a therapeutic strategy to target EMT-induced metastasis disease, and the targeting vimentin by IGFBP-3 contributing to our understanding of the better means to control cancer metastasis.

## Data Availability

The data presented in this study are available in the article or supplementary files.

## Supplementary Materials

The following are available online at https://www.mdpi.com/2072-6694/13/5/1041/s1, Figure S1: The Spearman correlation coefficient showing the relationship between the IGFBP3 mRNA expression and the mRNA expression of EMT markers in tumors derived from patients with lung cancer. Supplementary Figures: the whole western blot figures.
