# Supplementary material for: Insulin-Like Growth Factor Binding Protein-3 Exerts Its Anti-Metastatic Effect in Aerodigestive Tract Cancers by Disrupting the Protein Stability of Vimentin
Source: Cancers (Basel). 2021 Mar 2;13(5):1041. doi: 10.3390/cancers13051041 (PMC7958122; doi:10.3390/cancers13051041)

Full unedited gel for Figure 1A

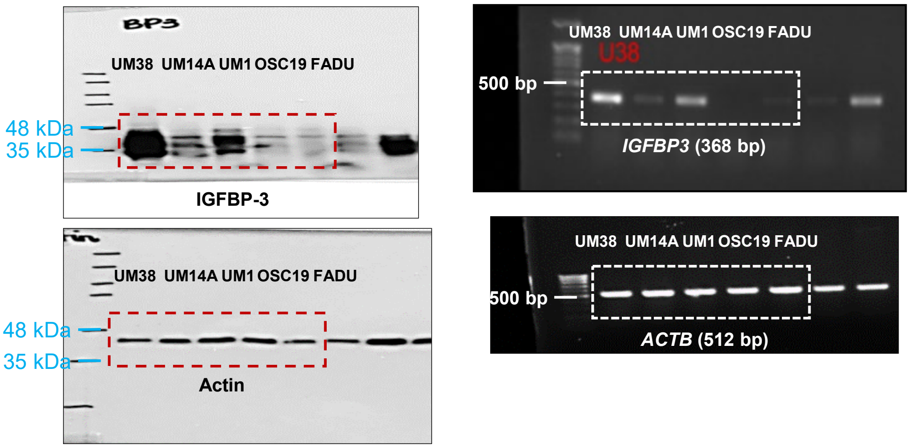

Full unedited gel for Figure 1B

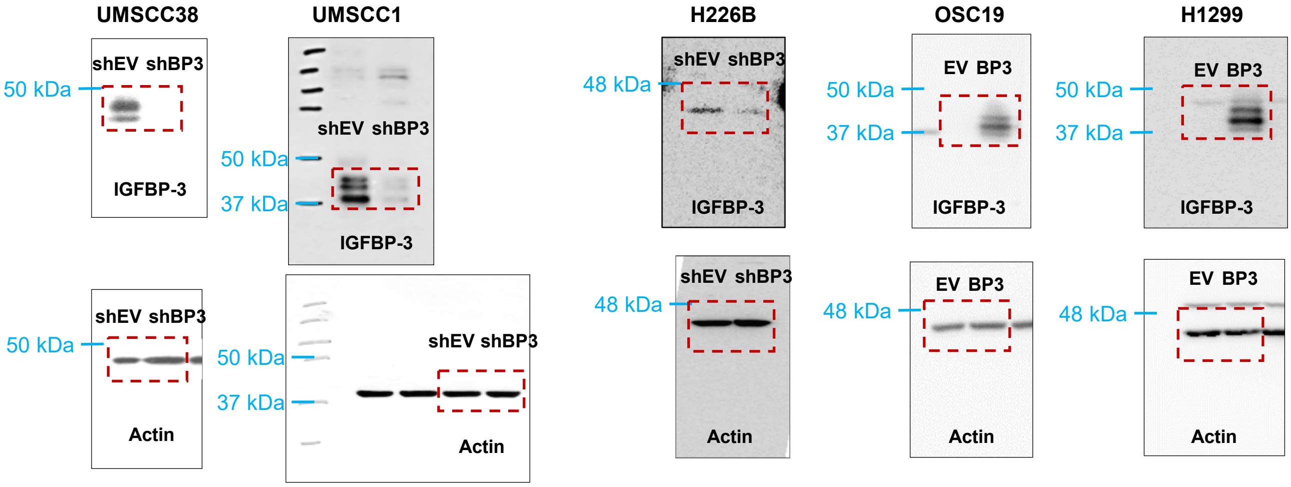

Full unedited gel for Figure 1G

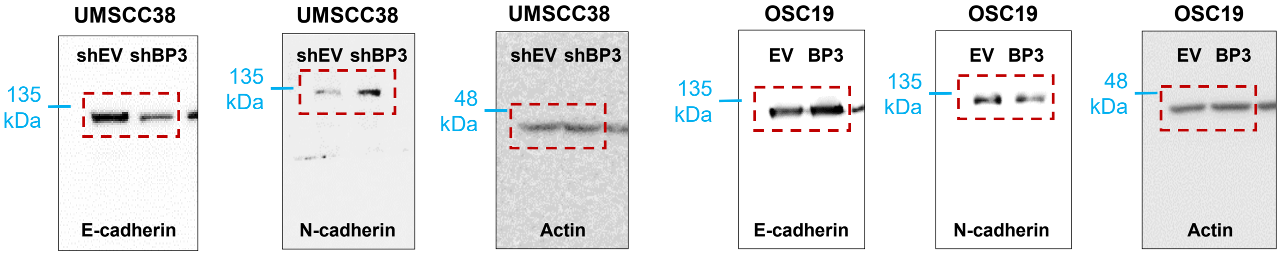

Full unedited gel for Figure 3B

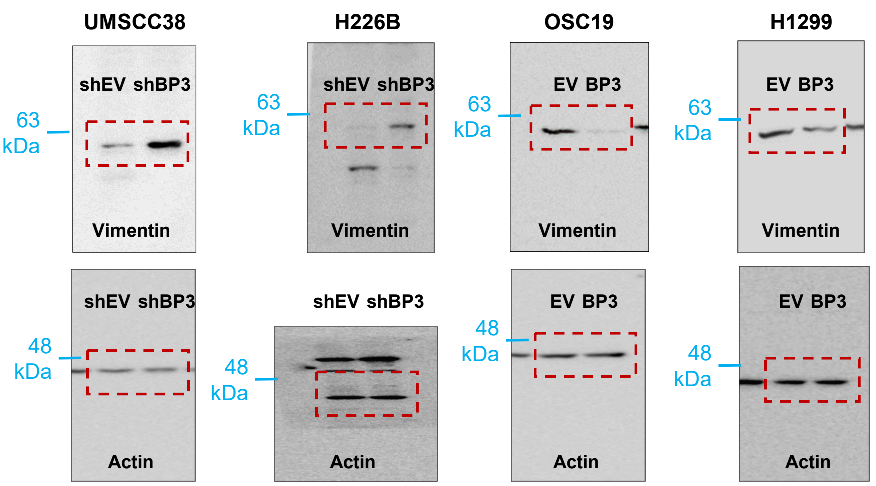

Full unedited gel for Figure 3D

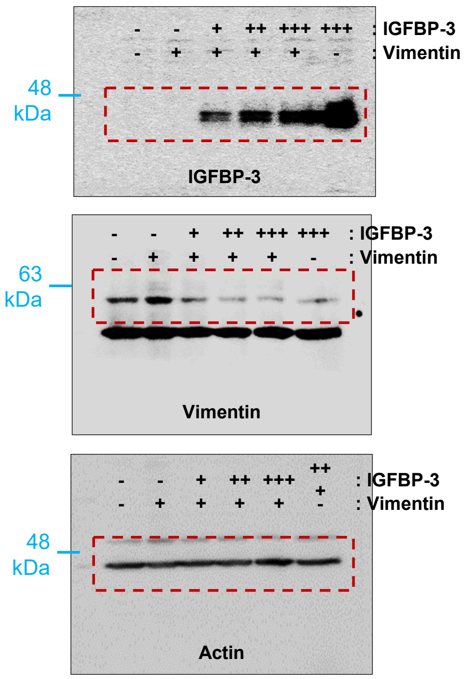

Full unedited gel for Figure 3F

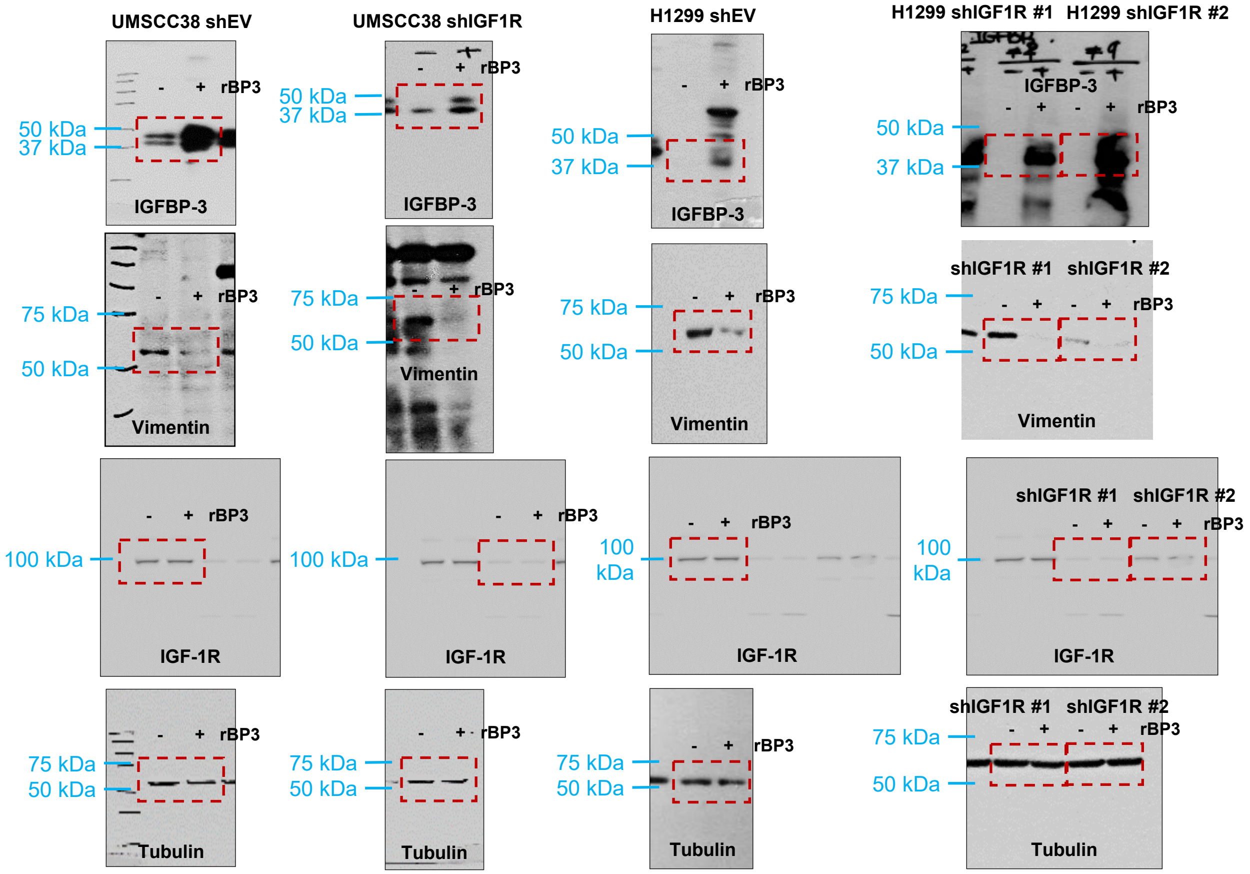

Full unedited gel for Figure 3G

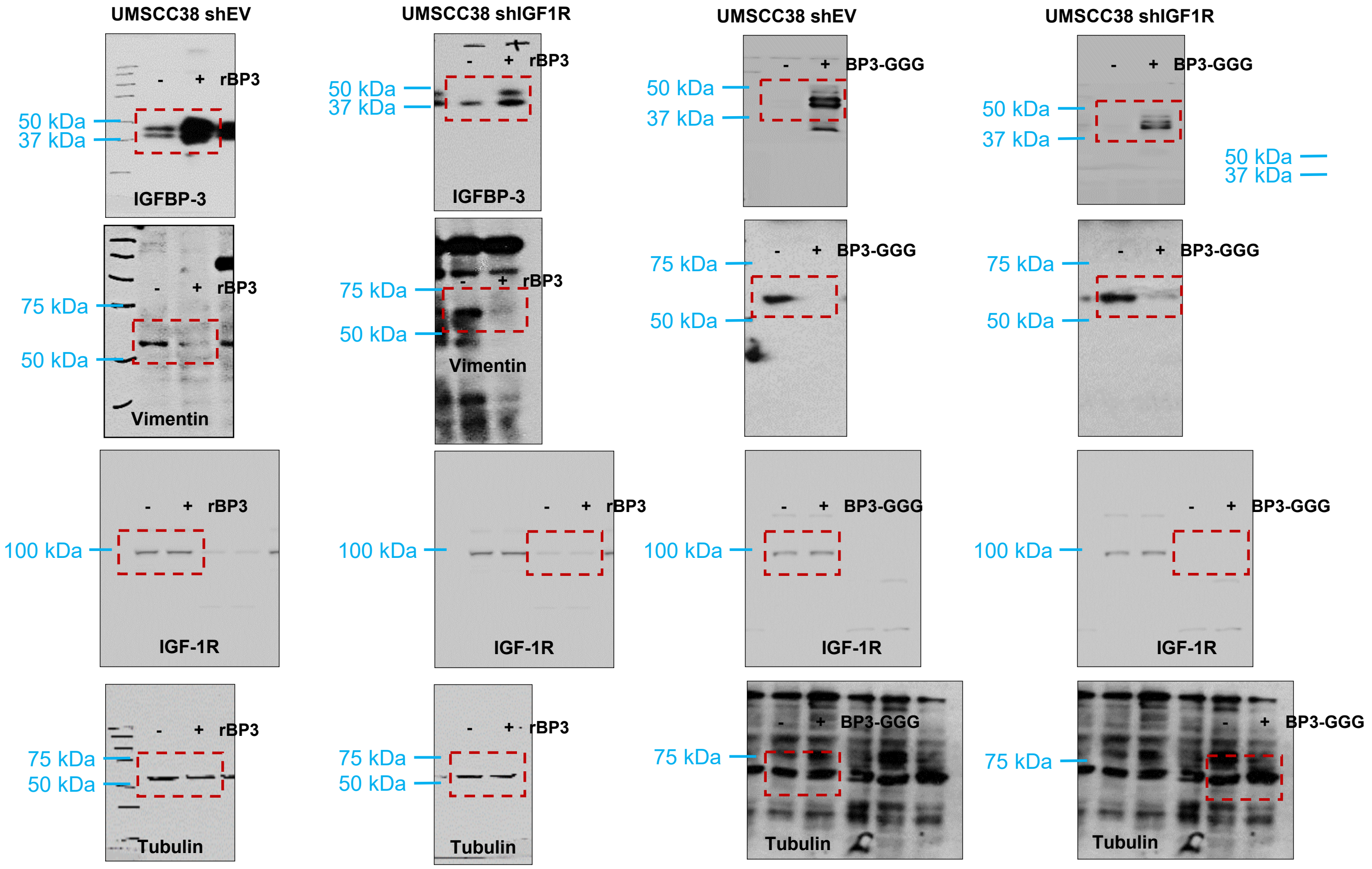

Full unedited gel for Figure 4B

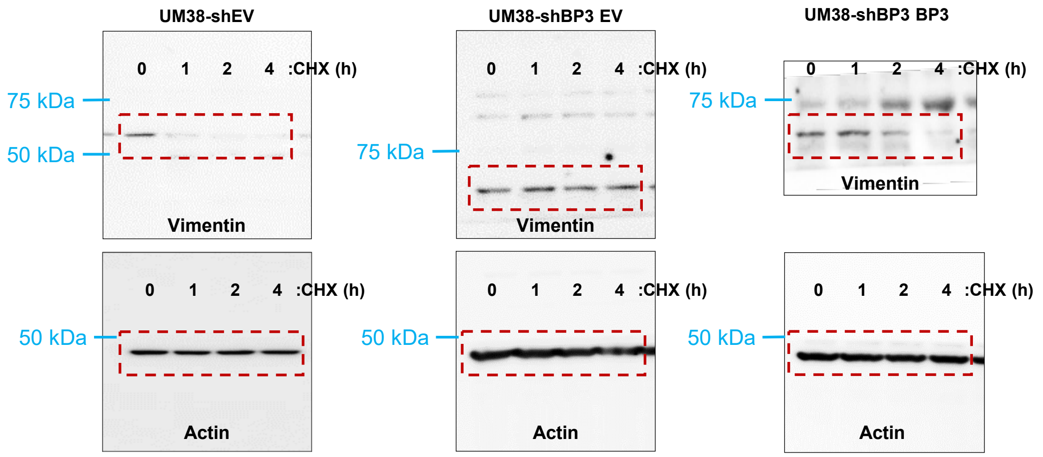

Full unedited gel for Figure 4D

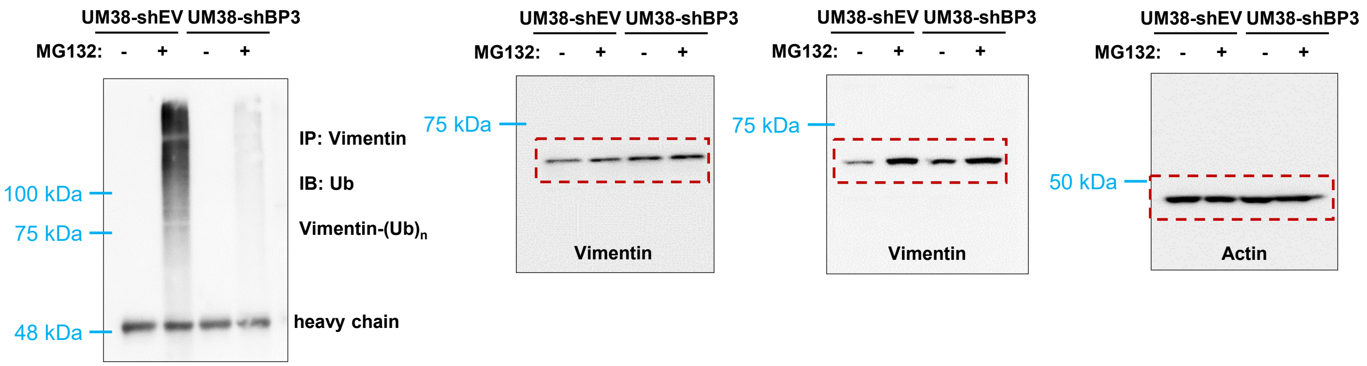

Full unedited gel for Figure 4E

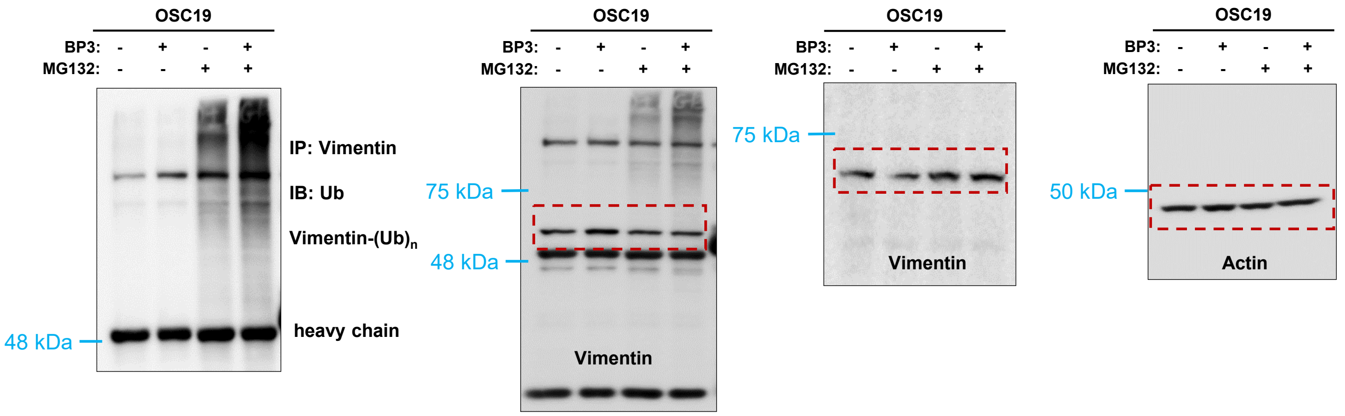

Full unedited gel for Figure 4F

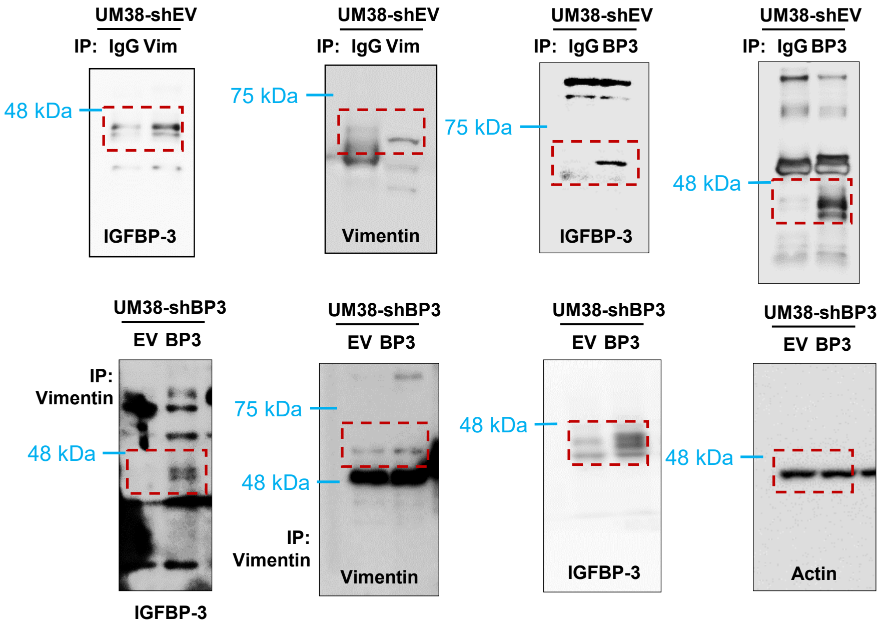

Full unedited gel for Figure 4G

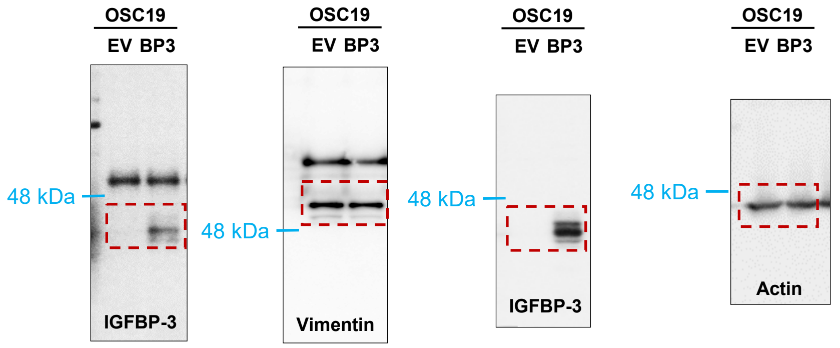

Full unedited gel for Figure 4H

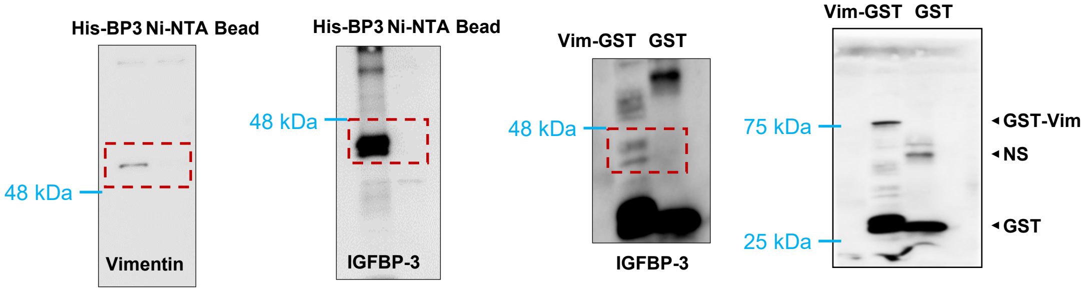

Full unedited gel for Figure 5A

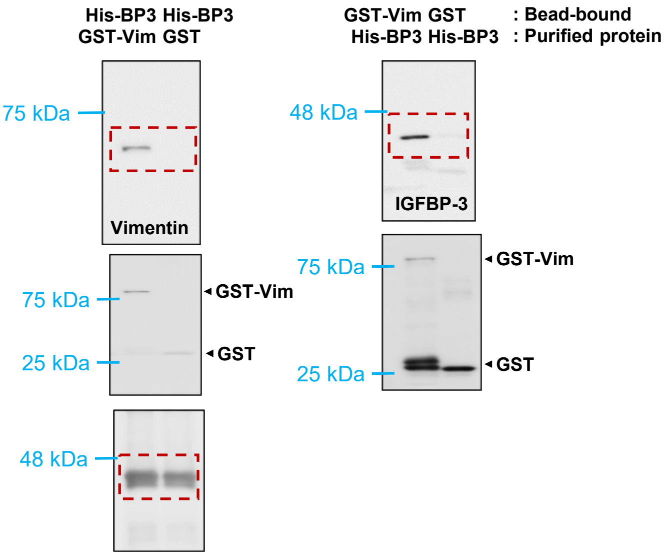

Full unedited gel for Figure 5C

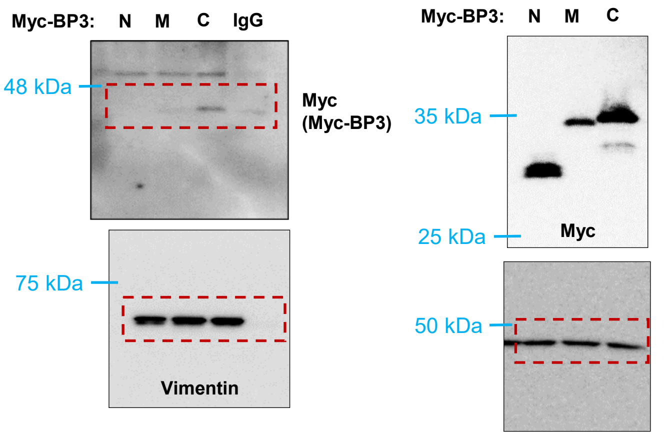

Full unedited gel for Figure 5D

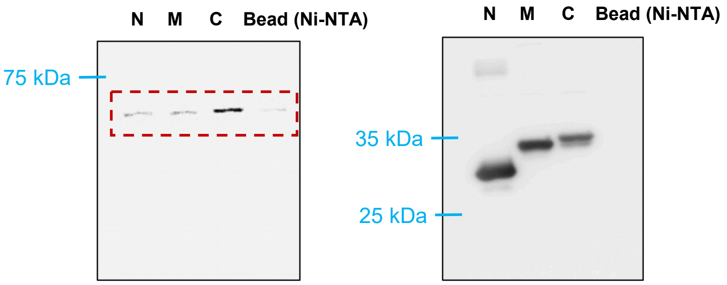

Full unedited gel for Figure 5E

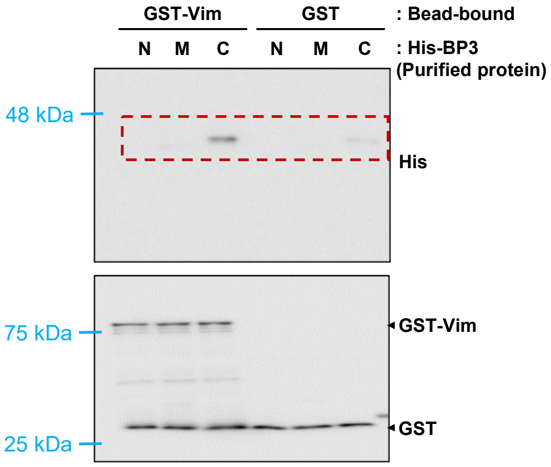

Full unedited gel for Figure 5F

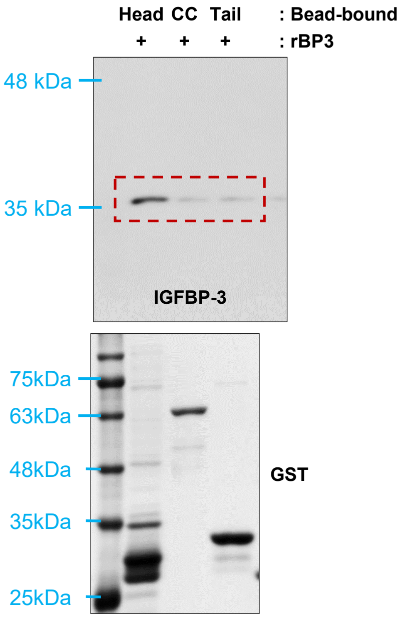

Full unedited gel for Figure 5G

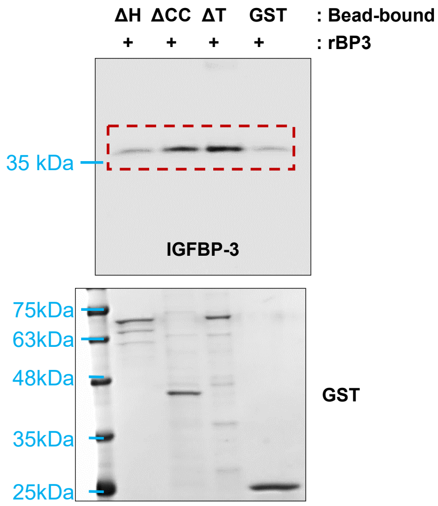

Full unedited gel for Figure 6A

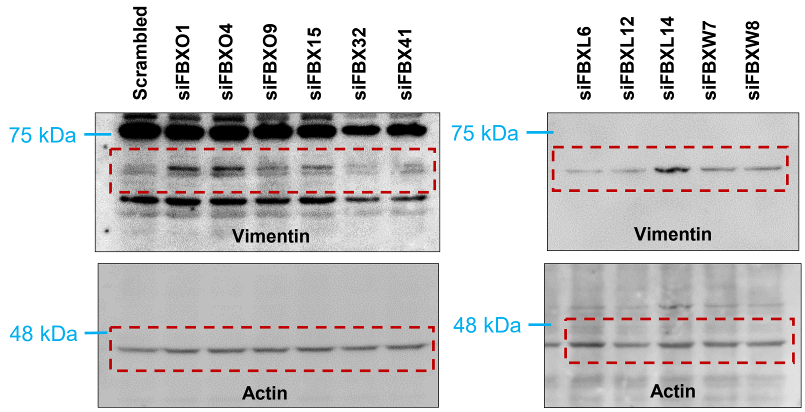

Full unedited gel for Figure 6B

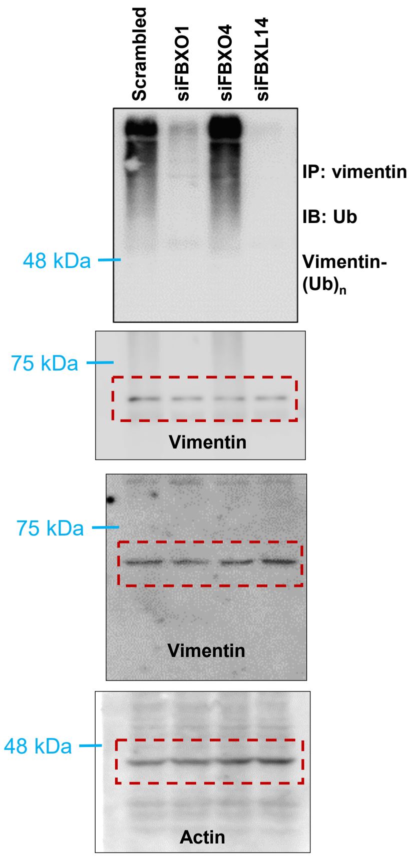

Full unedited gel for Figure 6C

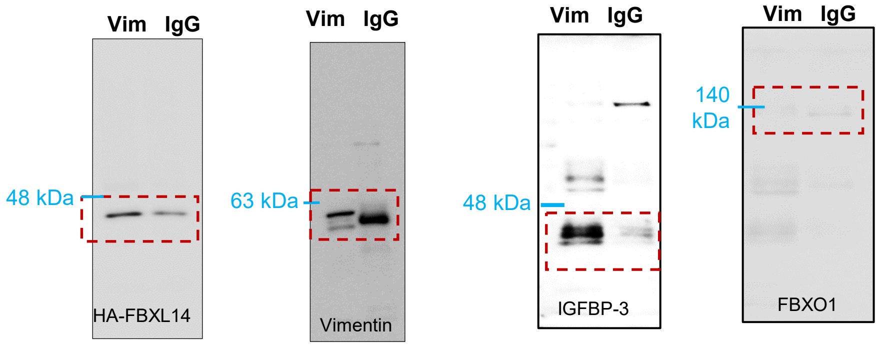

Full unedited gel for Figure 6D

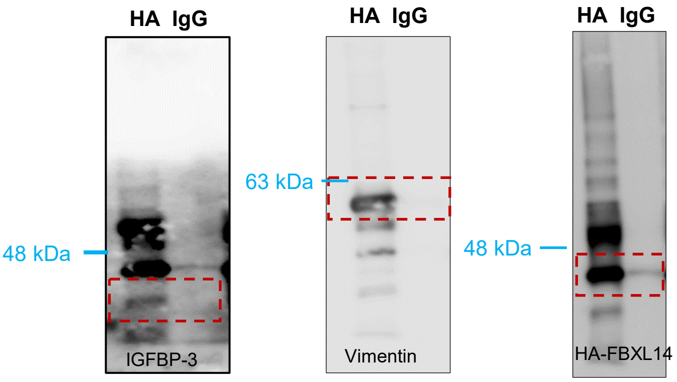

Full unedited gel for Figure 6E

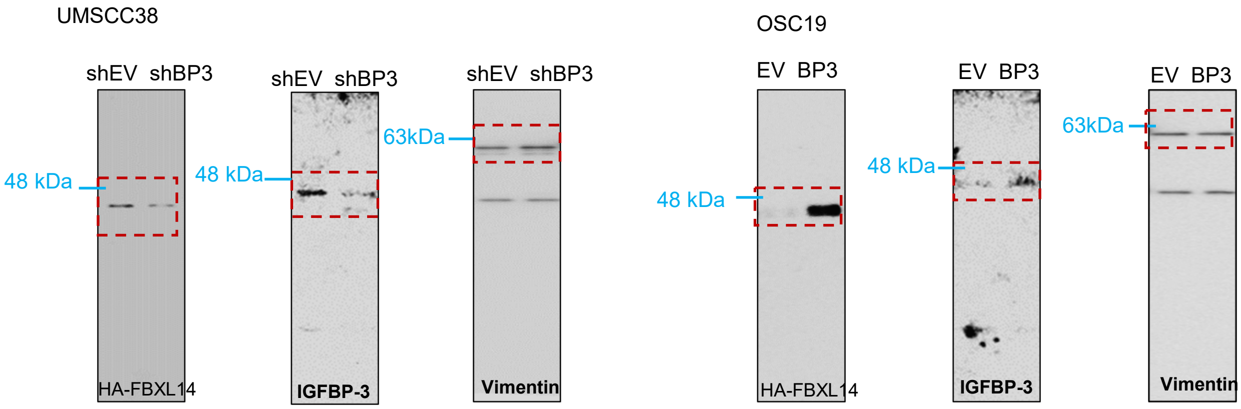

Full unedited gel for Figure 6F

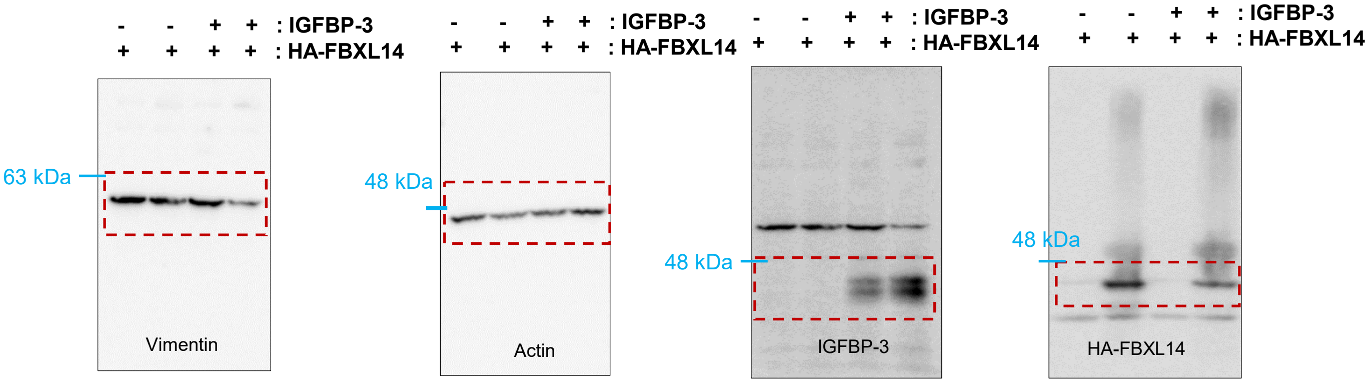

Full unedited gel for Figure 6G

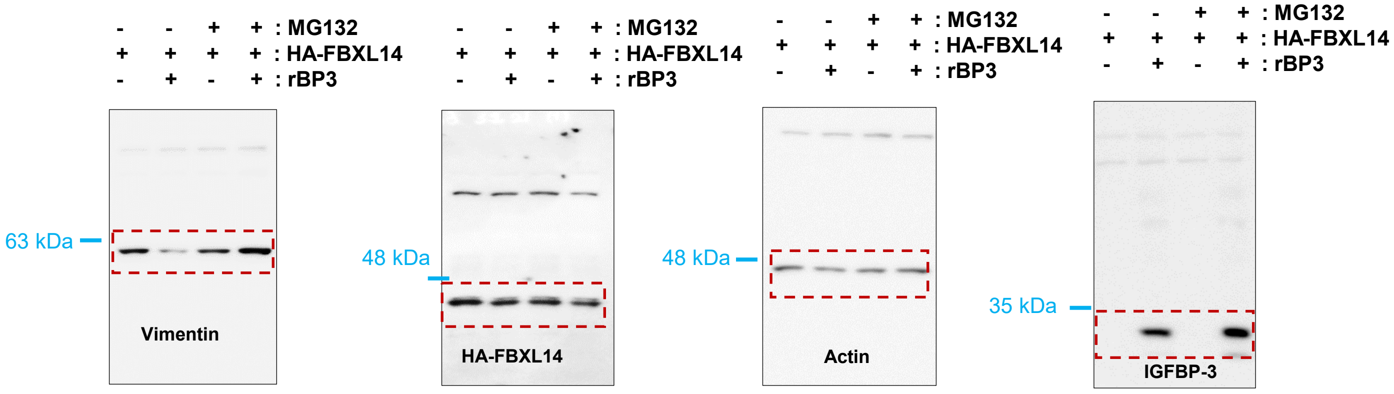

Full unedited gel for Figure 6H

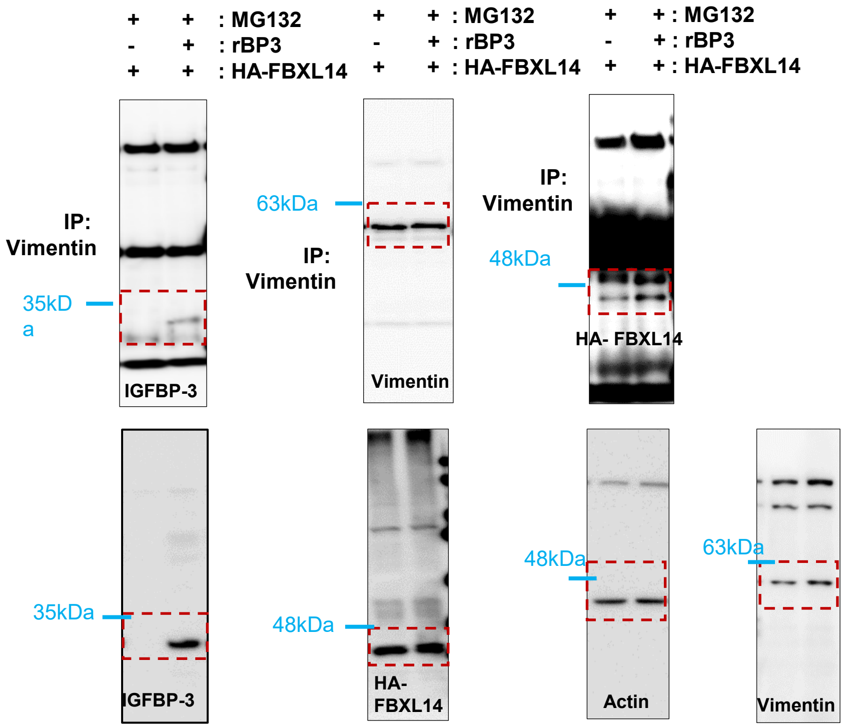

Full unedited gel for Figure 6I

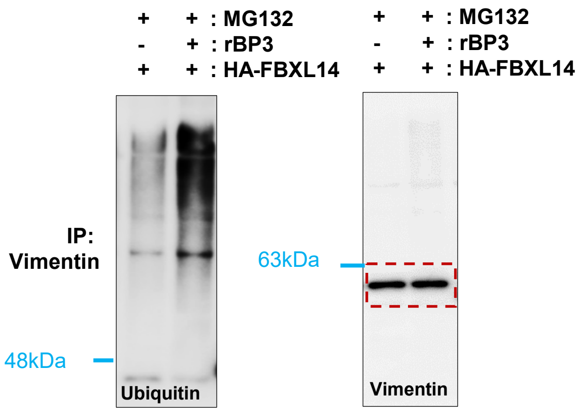

Full unedited gel for Figure 6J

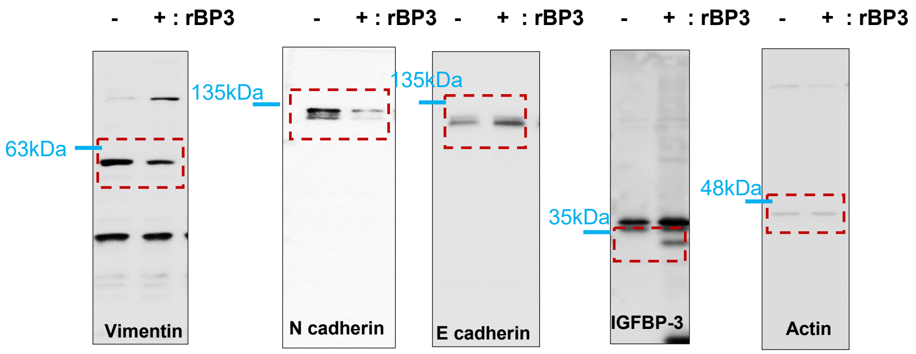

Supplement: Supplementary file 1 [file cancers-13-01041-s001.zip › cancers-1129783-original-images.pdf]
