# Supplementary material for: Insulin-Like Growth Factor Binding Protein-3 Exerts Its Anti-Metastatic Effect in Aerodigestive Tract Cancers by Disrupting the Protein Stability of Vimentin
Source: Cancers (Basel). 2021 Mar 2;13(5):1041. doi: 10.3390/cancers13051041 (PMC7958122; doi:10.3390/cancers13051041)
Supplement: Supplementary file 1 [file cancers-13-01041-s001.zip › cancers-1129783-supplementary.pdf]

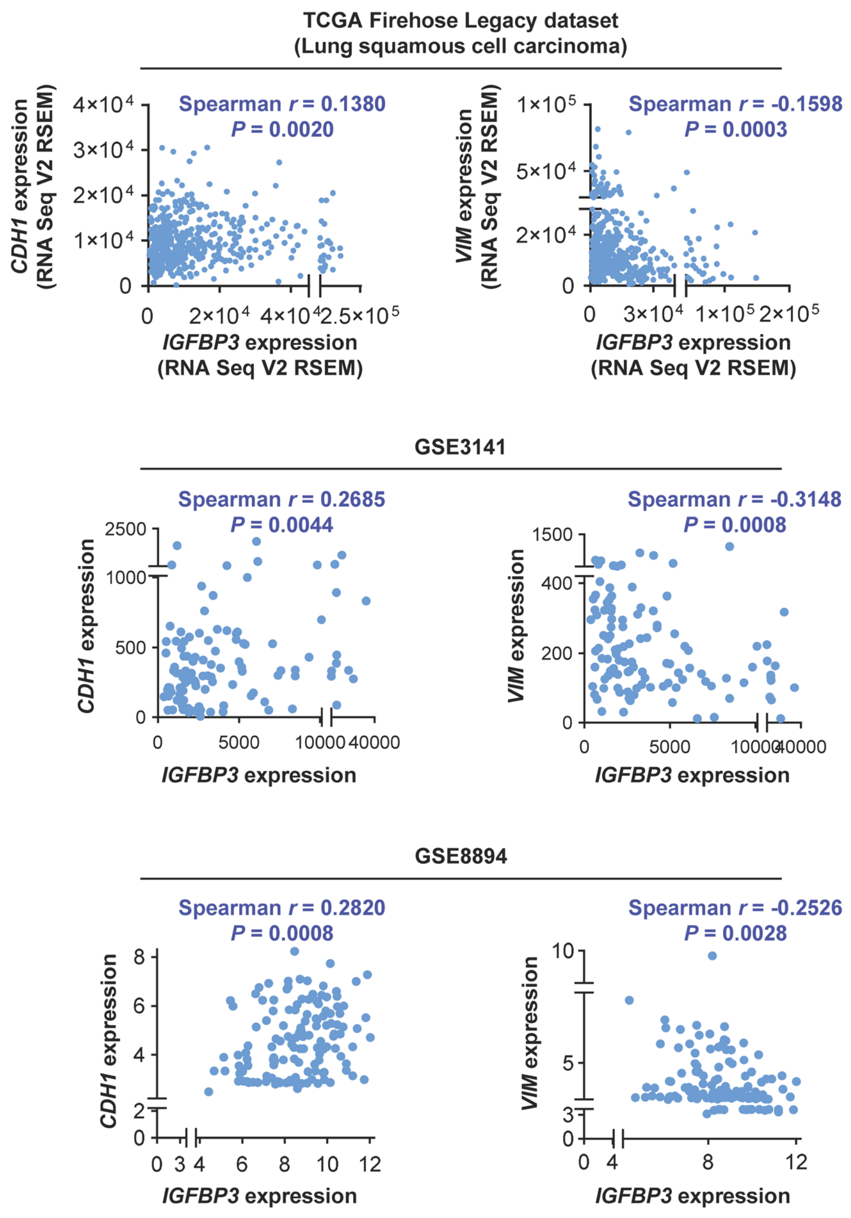

Figure S1. The Spearman correlation coefficient showing the relationship between the IGFBP3 mRNA expression and the mRNA expression of EMT markers in tumors derived from patients with lung cancer. The correlation was determined by using analysis of the TCGA Firehose Legacy dataset of lung squamous cell carcinoma (Top), the GSE3141 dataset (Middle), and the GSE8894 dataset (Bottom).
